# Exploratory assessment of the potential protective effects of Kampo treatment against low dose medical radiation: a single arm prospective observational study

**DOI:** 10.3389/fphar.2026.1888108

**Published:** 2026-08-07

**Authors:** Hongyang Li, Yuri Kawashima, Lian Liang, Che-Hong Chen, Ngo Quang Trung, Gloriamaris Loy Caraos, Yasuha Kinugasa, Kurumi Fujioka, Chiemi Sakai, Akihiko Adachi, Jiying Sun, Kazuo Awai, Yasushi Orihashi, Yasuki Kihara, Daria Mochly-Rosen, Satoshi Tashiro, Keiko Ogawa-Ochiai

**Affiliations:** 1 Kampo Clinical Center, Hiroshima University Hospital, Kasumi, Hiroshima, Japan; 2 Research Institute for Radiation Biology and Medicine, Hiroshima University, Hiroshima, Japan; 3 Department of Virology & Parasitology, Graduate School of Medicine, Osaka Metropolitan University, Osaka, Japan; 4 Department of Chemical and Systems Biology, Stanford University School of Medicine, Stanford, CA, United States; 5 Faculty of Traditional Medicine, Hai Phong University of Medicine and Pharmacy, Hai Phong, Vietnam; 6 Department of Cellular Biology, Research Institute for Radiation Biology and Medicine, Hiroshima University, Hiroshima, Japan; 7 Department of Science and Technology, Philippine Nuclear Research Institute, Quezon, Philippines; 8 Department of Cardiovascular Physiology and Medicine, Graduate School of Biomedical and Health Sciences, Hiroshima University, Hiroshima, Japan; 9 Department of Neurological Surgery, Chiba University, Chiba, Japan; 10 Department of Diagnostic Radiology, Hiroshima University Hospital, Hiroshima, Japan; 11 Clinical Research Center in Hiroshima, Hiroshima University Hospital, Hiroshima, Japan; 12 Department of Cardiovascular Medicine, Kobe City Medical Center General Hospital, Kobe, Japan

**Keywords:** chromosomal aberrations, D-limonene, functional dyspepsia, Kampo, low-dose computed tomography, peptide nucleic acid fluorescence *in situ* hybridization, radioprotective effect, γ-H2AX

## Abstract

**Introduction:**

Low-dose computed tomography (CT) scans can induce DNA damage, including chromosomal aberrations (CAs) and DNA double-strand breaks, which may contribute to long-term cancer risk. D-limonene, a bioactive component present in several Kampo formulas, has shown radioprotective effects in preclinical studies; however, clinical evidence remains limited, and the gastrointestinal side effects associated with isolated D-limonene may restrict its clinical application. This single-arm observational pilot study aimed to evaluate whether Kampo formulas containing D-limonene could mitigate CT-induced cytogenetic damage and to generate preliminary data for designing a future definitive trial.

**Methods:**

Thirty-nine patients with functional dyspepsia received one of four D-limonene-containing Kampo formulas: bukuryoin, rikkunshito, heiisan, or nichinto. Peripheral blood lymphocytes were collected before and after Kampo treatment and exposed in vitro to CT radiation. Chromosomal aberrations were assessed using peptide nucleic acid fluorescence in situ hybridization (PNA-FISH), while DNA double-strand breaks were evaluated by γ-H2AX immunofluorescence. Wilcoxon signed-rank tests and linear mixedeffects models were used to assess radiation effects and Kampo-related interactions, and intraclass correlation coefficients (ICCs) were calculated to compare assay reliability.

**Results:**

CT exposure significantly increased CAs before Kampo treatment (p = 0.024), whereas this increase was attenuated after treatment (p = 0.417). However, neither the pre–post difference-in-differences analysis (p = 0.428) nor the CT × Kampo interaction in the mixed-effects model (β = –0.72; 95% CI, –2.19 to 0.75; p = 0.343) reached statistical significance. γ-H2AX showed a similar trend. PNA-FISH demonstrated higher measurement reliability than γ-H2AX immunofluorescence (ICC = 0.89 vs. 0.65). Sample size estimation based on PNA-FISH indicated that 275–295 evaluable participants per arm would be required for a future definitive trial.

**Discussion:**

Overall, Kampo formulas containing D-limonene showed a trend toward mitigating CT-induced cytogenetic injury, and PNA-FISH appeared to provide a more reliable endpoint than γ-H2AX. These exploratory findings support further investigation in a randomized controlled trial to determine whether D-limonene-containing Kampo formulas can meaningfully mitigate CT-associated genotoxicity in humans.

## 1 Introduction

Computed tomography (CT) has become a cornerstone of modern medical imaging due to its non-invasive nature, precision, accuracy, and ability to detect early disease. Over recent decades, CT use has increased dramatically (Brenner, 2010). Although technological advances have reduced radiation doses per scan, the overall rise in CT utilization raises safety concerns (Garg et al., 2022; Miglioretti and Smith-Bindman, 2011). Multiple studies report an increased risk of leukemia and other cancer, particularly in children (Hauptmann et al., 2023), with more recent epidemiological evidence extending these risks to adults (Smith-Bindman et al., 2025).

Ionizing radiation from CT scans can induce DNA double-strand breaks, among the most deleterious forms of DNA damage (Schubert, 2021). Although repair mechanisms exist, errors may occur, leading to chromosomal aberrations (CAs) and elevated cancer risk. Spontaneous CAs in peripheral blood lymphocytes (PBLs) have been correlated with cancer incidence (Bonassi et al., 2008). Our previous studies similarly demonstrated that medical radiation from CT scans, even at doses below 100 mGy, can cause chromosomal abnormalities (Shi et al., 2018; Sakane et al., 2020). These findings emphasize the need for protective interventions to reduce the biological impact of low-dose CT exposure.

Radioprotective strategies have long been studied, ranging from synthetic agents to natural antioxidants (Lu et al., 2019; Cheng et al., 2019; Landauer et al., 2003; Hensley et al., 1999; Xu et al., 2021). Amifostine, a thiol compound and potent free radical scavenger, remains the only FDA-approved radioprotector in clinical use. However, its effectiveness is limited by dose-limiting side effects (Moulder, 2002; Hoffmann et al., 2001; Kligerman et al., 1984; Jagetia and Baliga, 2002). Given these limitations, research has shifted toward safer alternatives, with particular attention to natural products. Plant-derived metabolites are of special interest because they combine relative safety with diverse bioactive properties (Wang et al., 2020; Raj et al., 2022; Haritwal et al., 2022; Faramarzi et al., 2021). Botanical drugs are particularly appealing because they combine antioxidant, immunomodulatory, and other protective effects with favorable tolerability.

Kampo medicine, a traditional Japanese system based on multiple botanical drugs, has recently gained interest as a potential radioprotective approach. One of its major metabolites, D-limonene—a citrus-derived monoterpene present in formulas such as bukuryoin, rikkunshito, heiisan, and nichinto—activates aldehyde dehydrogenase (ALDH) and has been shown to reduce radiation-induced damage at high doses in experimental models (Saiki et al., 2018; Vigushin et al., 1998; Ning et al., 2012). Although gastrointestinal side effects limit its clinical use at pharmacological doses (Saiki et al., 2018), Kampo formulas contain D-limonene at milligram levels within multi-botanical drug formulations. Such combinations may mitigate low-dose radiation effects without major toxicity. However, their radioprotective potential against CT-induced genotoxicity in humans remains largely unexplored, leading us to hypothesize that Kampo formulas containing D-limonene could provide protective effects with improved tolerability.

Accurate evaluation of radiation-induced genotoxicity is essential for assessing medical radiation risks. Phosphorylated histone H2AX (γ-H2AX) is a sensitive marker of DNA double-strand breaks, forming nuclear foci at sites of damage (Löbrich et al., 2010; Grudzenski et al., 2010). Cytogenetic analysis of CAs in PBLs remains the gold standard for biological dosimetry, though it is labor-intensive (International Atomic Energy Agency (IAEA), 2011; Valentin, 2005). To address these limitations, we developed a fluorescence *in situ* hybridization method using centromere and telomere peptide nucleic acid probes (PNA-FISH), enabling efficient detection of unstable aberrations after medical radiation exposure (Shi et al., 2012; Imano et al., 2021). While both γ H2AX and PNA FISH are widely used biomarkers, no study has directly compared their suitability for clinical trial sample size estimation.

In this study, we investigated whether Kampo formulas could mitigate CT-induced genotoxicity in patients with functional dyspepsia (FD), a population commonly prescribed these formulas (Oshima, 2024). Using PNA-FISH and γ-H2AX assays in PBLs, we conducted a single-arm observational study to evaluate chromosomal and DNA damage before and after Kampo administration. A secondary objective was to compare the utility of PNA FISH or γ H2AX assays for estimating sample size in future trials.

## 2 Materials and methods

### 2.1 Study design and setting

**FIGURE 1:**
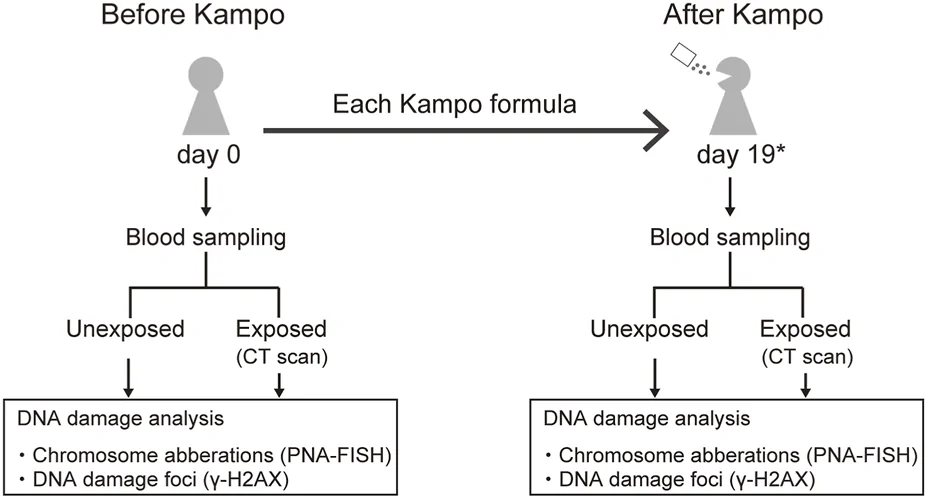
Schematic overview of the study design. An asterisk (*) indicates that the duration of Kampo treatment represents the average.

We conducted a single-arm, observational study at Hiroshima University Hospital between 2 September 2021, and 27 December 2023. FD Patients received a Kampo formula containing D-limonene, the primary component of Citrus reticulata Blanco [Rutaceae; Citri Unshiu Pericarpium]. Individuals without detectable organic gastrointestinal or hematological abnormalities were considered a surrogate healthy cohort. A schematic overview of the study design is shown in Figure 1. As this was an exploratory study, no formal sample size calculation was performed. The target sample size of approximately 40 participants was determined based on clinical feasibility and prior prospective studies in patients with functional dyspepsia (Gerson and Triadafilopoulos, 2005).

### 2.2 Patients

Patients were eligible if they were clinically diagnosed with FD based on the Rome IV criteria (Aziz et al., 2020) and were newly prescribed one of four Kampo formulas containing D-limonene (bukuryoin, rikkunshito, heiisan, and nichinto). The exclusion criteria were as follows: pregnancy, possibility of pregnancy, a history of abdominal surgery, diabetes mellitus, celiac disease or inflammatory bowel disease, active psychiatric conditions; current participation in another interventional clinical study; known hypersensitivity to any of the botanical drugs contained in the Kampo formulas and inability to provide informed consent or complete study procedures. To minimize bias from prior Kampo exposure, all patients were required to refrain from taking any Kampo formulas for at least 2 weeks before enrollment.

### 2.3 Research ethics

The study was conducted in accordance with the Declaration of Helsinki (Williams, 2008) and approved by the Ethics Committee of Hiroshima University (no. E2021-2483). Patients were fully informed about potential risks, and all provided written informed consent before participating. Consent procedures included signing a written form confirming their understanding by the study protocol and willingness to participate. Participants were free to withdraw from the trial at any time.

### 2.4 Medication

The average treatment duration with Kampo formulas was 19 consecutive days, with variations based on patients’ symptoms and the specific formulas prescribed by Kampo physicians. The prescribed Kampo formulas include bukuryoin, rikkunshito, heiisan and nichinto were manufactured by Tsumura & Co. (Tokyo, Japan) and approved for medical use by the Ministry of Health, Labour and Welfare (MHLW) of Japan. Each formula was prepared from crude botanical drugs through standardized hot-water extraction, followed by solid-liquid separation, concentration, spray-drying, and granulation according to Good Manufacturing Practice (GMP)-compliant manufacturing procedures. Their manufacturing methods, components, and quantities comply with the standards of the Japanese Pharmacopoeia (The Japanese Pharmacopoeia, 18th edition, English version, 2021). The Japanese Pharmacopoeia is the official compendium issued by the MHLW, which defines the origins and characteristics of listed botanical drugs and Kampo formulas, and specifies their permissible limits, purity tests, and analytical methods to ensure quality, safety, and consistency. Detailed information on each formula can be accessed through the STORK database (http://mpdb.nibiohn.go.jp/stork/) (accessed on 3 February 2026), managed by the National Institutes of Biomedical Innovation, Health and Nutrition (NIBN). The compositions of the Kampo formulas are delineated in Table 1. Pharmaceutical characteristics, extraction procedures, drug–extract ratios (DERs), and quality-control information are summarized in Supplementary File 1. Manufacturer-provided HPLC fingerprint chromatograms and supporting quality-control documentation are available in Supplementary File 2. The Kampo formulas used in this study were commercially available pharmaceutical products manufactured in Japan and approved for clinical use. No wild-collected or CITES-listed species were used, and the products complied with all applicable national regulations.

**TABLE 1:** Botanical drug composition of Kampo formulas used in the study.

Botanical drugs	Scientific name	Bukuryoin	Rikkunshito	Heiisan	Nichinto
Poria sclerotium	Wolfiporia cocos ryvarden et gilbertson [polyporaceae; poria sclerotium]	5.0 g	4.0 g	​	5.0 g
Atractylodes lancea rhizome	Atractylodes lancea de candolle; atractylodes chinensis koidzumi [asteraceae; atractylodes lancea rhizome]	4.0 g	4.0 g	4.0 g	​
*Citrus unshiu peel	Citrus unshiu markowicz; citrus reticulata blanco [rutaceae; citri unshiu pericarpium]	3.0 g	2.0 g	3.0 g	4.0 g
Ginseng	Panax ginseng C. A. Meyer [araliaceae; ginseng radix et rhizoma]	3.0 g	4.0 g	​	​
Immature orange	Citrus aurantium linné var. daidai Makino, Citrus aurantium linné; citrus natsudaidai hayata [rutaceae; aurantii fructus immaturus]	1.5 g	​	​	​
Ginger	Zingiber officinale roscoe [zingiberaceae; zingiberis rhizoma]	1.0 g	0.5 g	0.5 g	1.0 g
Pinellia tuber	Pinellia ternata breitenbach [araceae; pinellia tuber]	​	4.0 g	​	5.0 g
Jujube	Ziziphus jujuba miller var. inermis rehder [rhamnaceae; ziziphi fructus]	​	2.0 g	2.0 g	​
Glycyrrhiza	Glycyrrhiza uralensis fischer; glycyrrhiza glabra linné [fabaceae; glycyrrhizae radix et rhizoma]	​	1.0 g	1.0 g	1.0 g
Magnolia bark	Magnolia obovata thunb.; magnolia officinalis rehder et E. H. Wilson; magnolia officinalis var. biloba rehder et E. H. Wilson [magnoliaceae; magnoliae cortex]	​	​	3.0 g	​

* Containing D-Limonene.

The Kampo formulas used in this study are approved prescription medicines in Japan and were administered according to the approved package inserts. Known adverse reactions, drug interactions, precautions, and safety information were reviewed prior to study initiation. Participants were monitored for adverse events throughout the study period. Individuals with known hypersensitivity to any component of the Kampo formulas were excluded from participation. Concomitant medications prescribed for underlying medical conditions were permitted and recorded throughout the study period. Because this was an observational study conducted in routine clinical practice, no formal adherence assessment was performed. Medication use was confirmed through routine clinical interviews during follow-up visits.

### 2.5 Variables

In this pilot study, CAs were assessed by PNA-FISH analysis and expressed as frequency per 1,000 metaphase cells. γ-H2AX foci formation was evaluated as an additional marker of early DNA damage response. Patient demographic data, type of Kampo formulas, and Citri Unshiu Pericarpium (source of D-limonene) content were considered for subgroup analyses.

### 2.6 ALDH enzymatic assay

We extracted 200 mg of Kampo powder in 400 µL of DMSO overnight. After extraction, 2 µL of the soluble supernatant from each preparation was used in ALDH3A1 enzyme activity assays. Each assay contained 10 µg of purified recombinant human ALDH3A1 protein and followed a standard ALDH activity assay protocol (Saiki et al., 2018). Enzyme activity was expressed as percentage relative to assays using DMSO as the control. No positive control was included in this essay; therefore, the results should be regarded as qualitative and exploratory in nature.

### 2.7 *In vitro* radiation exposure

Heparinized peripheral blood samples (5 mL × 3) were collected from patients. One sample was left unexposed (control), while another was irradiated using a 320-detector CT scanner (Aquilion ONE, Toshiba Medical Systems Corp., Otawara, Japan) in combination with a human body phantom (Magen Phantom BMU-1, Kyoto Kagaku, Kyoto, Japan). The phantom was used to simulate human body attenuation and scattering conditions during CT exposure. Heparinized blood samples were placed in syringes and inserted into the center hole of the phantom during irradiation. The irradiation protocol was set at a dose-length product of up to 1,600 mGy·cm, equivalent to 100 mGy.

### 2.8 PNA-FISH and γ-H2AX analysis

All experiments were conducted according to previously described procedures (Loy-Caraos et al., 2025) and are detailed in the Supplementary File 3. We calculated unstable CAs, specifically dicentric and ring chromosomes, as indicators of CAs.

### 2.9 Statistical analyses

The primary analysis compared data obtained before and after Kampo treatment (bukuryoin, rikkunshito, heiisan, or nichinto). Subgroup analyses were conducted by (i) Kampo formula and (ii) Citri Unshiu Pericarpium content. Since only one patient received nichinto, this group was excluded. For the component-based analysis, patients were stratified by daily intake of Citri Unshiu Pericarpium (source of D-limonene): ≥3.0 g vs. < 3.0 g. Baseline characteristics among the three groups were compared using Kruskal–Wallis test for continuous variables, and the Fisher’s exact test for categorical variables, as appropriate.

Wilcoxon signed-rank test was performed for pairwise comparisons. To account for repeated measures (chromosomal abnormalities before vs. after treatment in the same individuals), a linear mixed-effects model (LMM) was applied. The LMM included Kampo treatment (before vs. after), CT exposure (exposed vs. unexposed), and their interaction (Kampo × Exposure) as fixed effects, with patient ID as a random effect to account for within-subject correlations (compound symmetry). Model estimation was conducted using restricted maximum likelihood. All statistical analyses were performed using R version 4.4.3. No multiplicity adjustment was applied because subgroup analyses were prespecified exploratory analyses intended to generate hypotheses rather than establish confirmatory evidence.

### 2.10 Sample size estimation for future clinical trial

We estimated the required sample size for paired two group comparisons using (i) a two level random intercept LMM and (ii) the Wilcoxon signed rank test, with a two-sided α = 0.05 and power 1−β = 0.80. The LMM (fit with lme4 in R) included a subject specific random intercept with variance $\sigma_{between}^{2}$ and residual with variance $\sigma_{within}^{2}$ under a compound-symmetry structure.

For the formula-based calculation, we approximated the LMM-derived effect as a within subject mean difference between exposed and unexposed conditions and applied the paired t-test formula in R (stats::power.t.test, type = “paired”). Because random intercepts cancel in within subject contrasts, the standard deviation for power was based only on the residual component: ${\text{SD}}_{diff}=\sqrt{2}\sigma_{within}$ , where $\sigma_{within}$ was taken from the LMM residual SD. For descriptive purposes, we also reported the ICC calculated as

$$ICC=\frac{\sigma_{between}^{2}}{\sigma_{between}^{2}+\sigma_{within}^{2}}$$

following recommendations for LMM. The ICC was not used in power calculations (Nakagawa et al., 2017; Lorah, 2018).

For the Wilcoxon-based calculation, the effect size was defined as the mean change in the exposure contrast from before to after Kampo treatment. The same t-test approximation (normal approximation to the signed-rank test) was applied, using the pooled SD calculated from the observed variances before and after treatment.

## 3 Results

### 3.1 Patients and baseline characteristics

**FIGURE 2:**
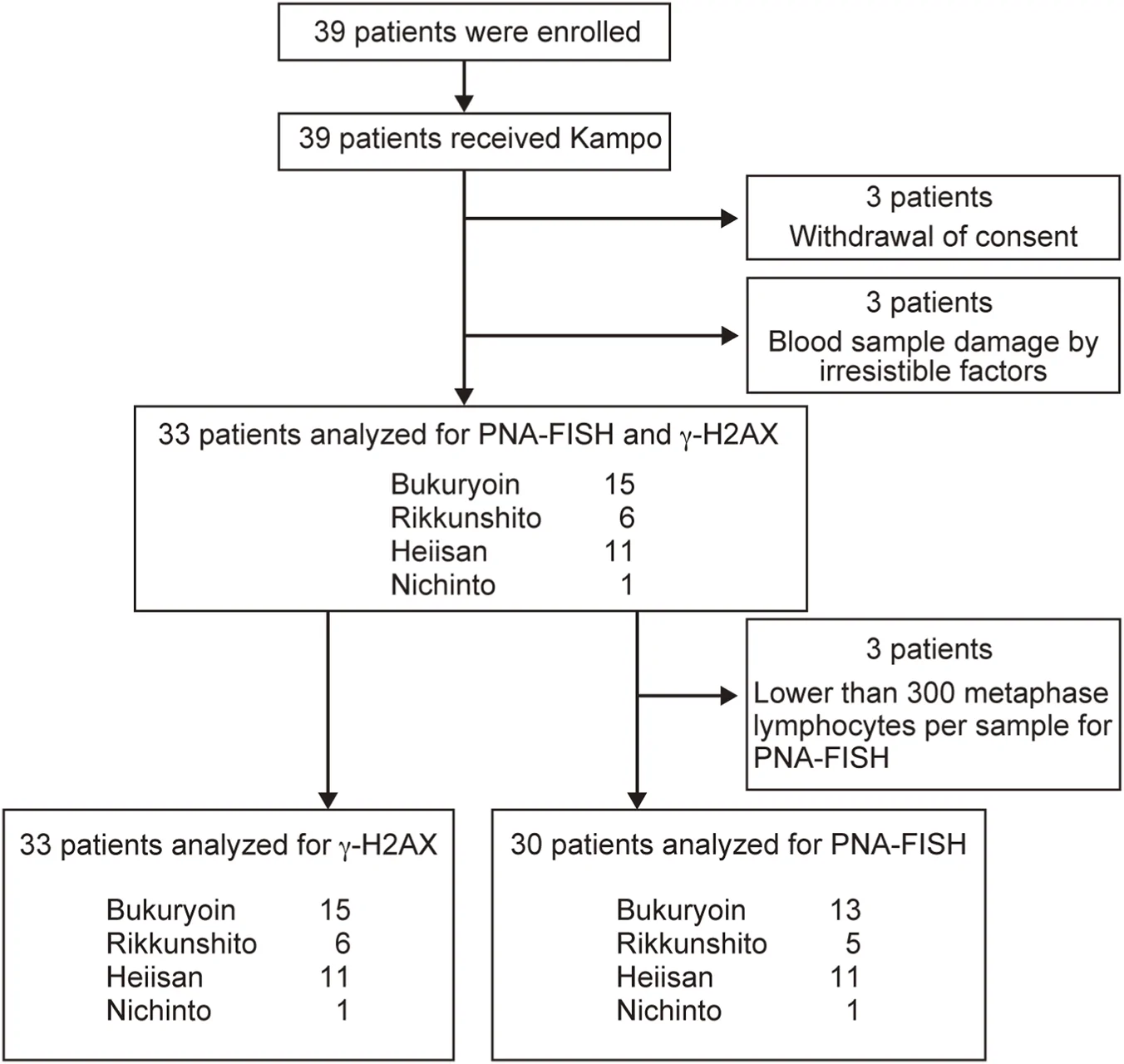
Flowchart of patient disposition.

Demographic data for the total 39 enrolled patients (mean age 54.8 ± 20.2; 16 males, 23 females) are shown in Table 2. Detailed individual patient information is provided in Supplementary File 4. The number of patients prescribed each of the four Kampo formulas is shown in Table 3. Among them, 33 patients were analyzed for *γ*-H2AX, and 30 were analyzed for PNA-FISH. Participant flow and reasons for exclusions are summarized in Figure 2. No significant differences were observed in baseline age, sex distribution, weight, height and baseline γ-H2AX foci among the bukuryoin, rikkunshito, and heiisan groups (all p > 0.05; Supplementary Table S1).

**TABLE 2:** Patient background characteristics (n = 39).

Items	Number	Mean ± SD
Age	​	54.8 ± 20.2
Height (cm)	​	161.4 ± 8.1
Weight (kg)	​	55.1 ± 10.7
BMI	​	21.1 ± 3.5
Sex		
Male	16	​
Female	23	​

**TABLE 3:** Number of patients taking each Kampo formula.

Kampo formula	Number
Bukuryoin	20
Rikkunshito	7
Heiisan	11
Nichinto	1

### 3.2 Radioprotective effects evaluated by frequency of CAs

We first evaluated the *in vitro* activity of Kampo formulas containing D-limonene. Bukuryoin, rikkunshito, heiisan, and nichinto all showed activation of ALDH3A1 using purified recombinant human protein (Supplementary Figure S1), confirming that these Kampo formulas contained sufficient D-limonene to assess *in vitro* activity.

**FIGURE 3:**
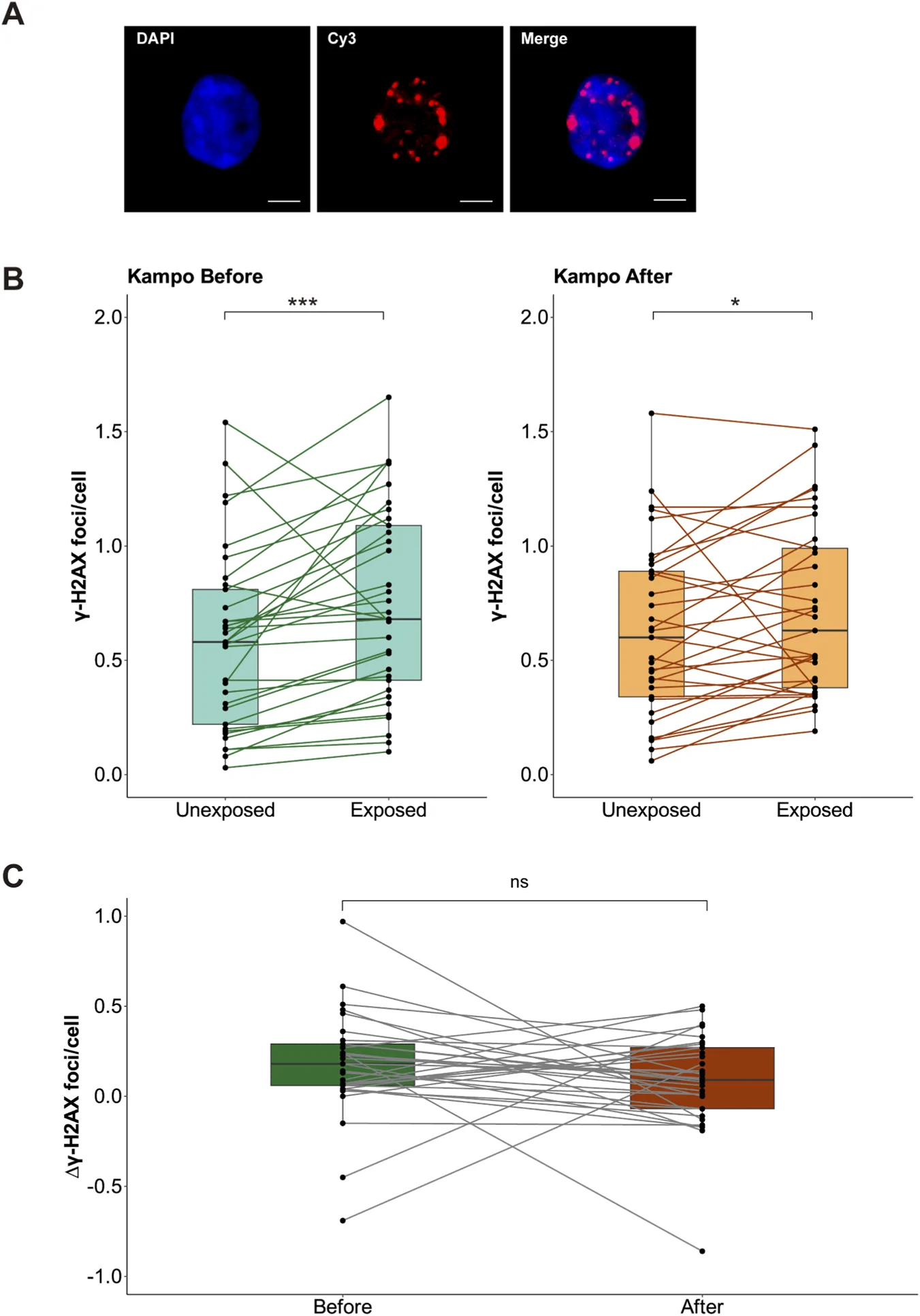
Analysis of CAs in FD patients before and after Kampo treatment. **(A)** Representative images of PNA-FISH in metaphase PBLs. DNA was stained with DAPI (blue); telomeres and centromeres were labeled with Cy3 (red) and FITC (green), respectively. Dicentric (arrowhead) and ring (arrow) chromosomes are indicated in the merged image. **(B)** Changes in CAs by CT exposure before (left) and after (right) Kampo treatment (before; p = 0.023, after; p = 0.428). **(C)** Comparison of CT-induced increase in CAs (exposed–unexposed) before and after Kampo treatment (p = 0.428). A total of 30 patients treated with bukuryoin, rikkunshito, heiisan or nichinto were included. Wilcoxon matched-pairs signed-rank test was used to compare each group. *p < 0.05; ns, not significant.

Next, we examined the effects of radiation exposure on CAs using PNA-FISH analysis (Figure 3A). The median difference (Δ) between unexposed and exposed samples before Kampo treatment was 0.77 (IQR, −0.26 to 3.47; Figure 3B left; Table 4, Supplementary Tables S2, S3), which was statistically significant (p = 0.023), consistent with our previous reports (Sakane et al., 2020; Lu et al., 2019). After Kampo treatment, this difference was attenuated to non-significance (Δ = 0.66, IQR, −1.50 to 1.52; p = 0.417; Figure 3B right; Supplementary Tables S4, S5), a pattern consistent with a radioprotective effect. Despite this attenuation, the pre–post change in the between-group difference (difference-in-differences) had a median value of −0.54 (IQR, −4.54 to 2.95, p = 0.428), which was not statistically significant (Figure 3C; Table 4). To further evaluate the CT induced effect and its modification by Kampo treatment, we fitted LMM accounting for the repeated measures design. Between the two candidate covariance structures (compound symmetry vs. unstructured), the compound symmetry model yielded a lower AICc (compound symmetry: 624.7 vs. unstructured: 629.6). The ICC was estimated at 0.89, supporting the assumption of equal correlations.

**TABLE 4:** Comparison of CT exposure-related changes in CAs before and after Kampo treatment.

Group	n	Median (IQR)	Mean ± SD
All patients	30	−0.54 (−4.54, 2.95)	−0.72 ± 5.20
Kampo formula subgroup			
Bukuryoin	13	−1.75 (−5.05, −0.17)	−1.61 ± 5.49
Rikkunshito	5	1.10 (−0.11, 4.87)	2.95 ± 5.24
Heiisan	11	−0.40 (−5.58, 2.77)	−1.46 ± 4.75
Component subgroup			
Citri unshiu pericarpium			
≧3 g	25	−1.33 (−5.56, 2.41)	−1.45 ± 4.97
<3 g	5	1.10 (−0.11, 4.87)	2.95 ± 5.24

Although the point estimate for the CT × Kampo interaction was negative (β = −0.72), the effect was not statistically significant (95% CI, −2.19 to 0.75; p = 0.343; Table 5). Taken together, these results suggest potential radioprotective effects of Kampo formulas containing D-limonene.

**TABLE 5:** Comparison of fitted data of LMM model and Wilcoxon matched pair singed rank test of PNA-FISH data.

Group	n	LMM	​	​	Wilcoxon signed rank
		Kampo × exposure interaction effect (95% CI)	p-value	​	p-value
All patients	30	−0.72 (−2.19, 0.75)	0.343	​	0.428
Kampo formula subgroup					
Bukuryoin	13	−1.61 (−4.17, 0.95)	0.232	​	0.244
Rikkunshito	5	2.95 (−0.48, 6.38)	0.133	​	0.438
Heiisan	11	−1.46 (−3.46, 0.53)	0.169	​	0.413
Component subgroup					
Citri unshiu pericarpium					
≧3 g	25	−1.45 (−3.04, 0.14)	0.079	*	0.428
<3 g	5	2.95 (−0.48, 6.38)	0.133	​	0.413

* p < 0.1.

### 3.3 Subgroup analysis of frequency of CAs

The total patient cohort was analyzed using two distinct subgroup classifications. The first subgroup was based on the type of Kampo formula. Since only one patient received nichinto in this study, we performed subgroup analyses for patients treated with bukuryoin, rikkunshito, or heiisan.

Before bukuryoin administration, the difference between unexposed and exposed groups in CAs was significant (Δ = 1.35, IQR, 0.39 to 3.50; p = 0.040: Supplementary Figure S2A; Table 4; Supplementary Tables S2, S3). After bukuryoin treatment, this difference was attenuated to non-significance (Δ = 0.47, IQR, −1.57 to 1.56; p = 0.735; Supplementary Figure S2A; Table 4; Supplementary Tables S2, S3), and the pre–post change in the between-group difference (difference in differences) was not statistically significant (Δ = −1.75, IQR, −5.05 to −0.17; p = 0.244; Supplementary Figure S2B; Table 4; Supplementary Tables S2, S3). This trend was consistent with the overall data from all 30 patients (Figures 3B,C). By contrast, a similar attenuation was not observed in rikkunshito or heiisan (Supplementary Figure S2C; Table 4; Supplementary Tables S2, S3). No statistically significant results were observed in the LMM analysis (Table 5).

The second subgroup classification was based on the amount of Citri Unshiu Pericarpium (containing D-limonene), dividing patients into those receiving ≥3.0 g/day (bukuryoin, heiisan, nichinto; 25 patients) and those receiving <3.0 g/day (rikkunshito; 5 patients). Although the pre-post change in the between-group difference was not statistically significant (Δ = −1.33, IQR, −5.56 to 2.4; p = 0.428; Table 4), the CT × Kamp interaction was estimated by the LMM was −1.45 (95% CI: −3.04 to 0.14, p = 0.079), directionally consistent with a radioprotective effect, though only marginally significant (Table 5). These results suggest a possible association between D-limonene and radioprotective effects and highlight the benefit of robust analytical methods.

### 3.4 Quantification of the number of γ-H2AX foci

**FIGURE 4:**
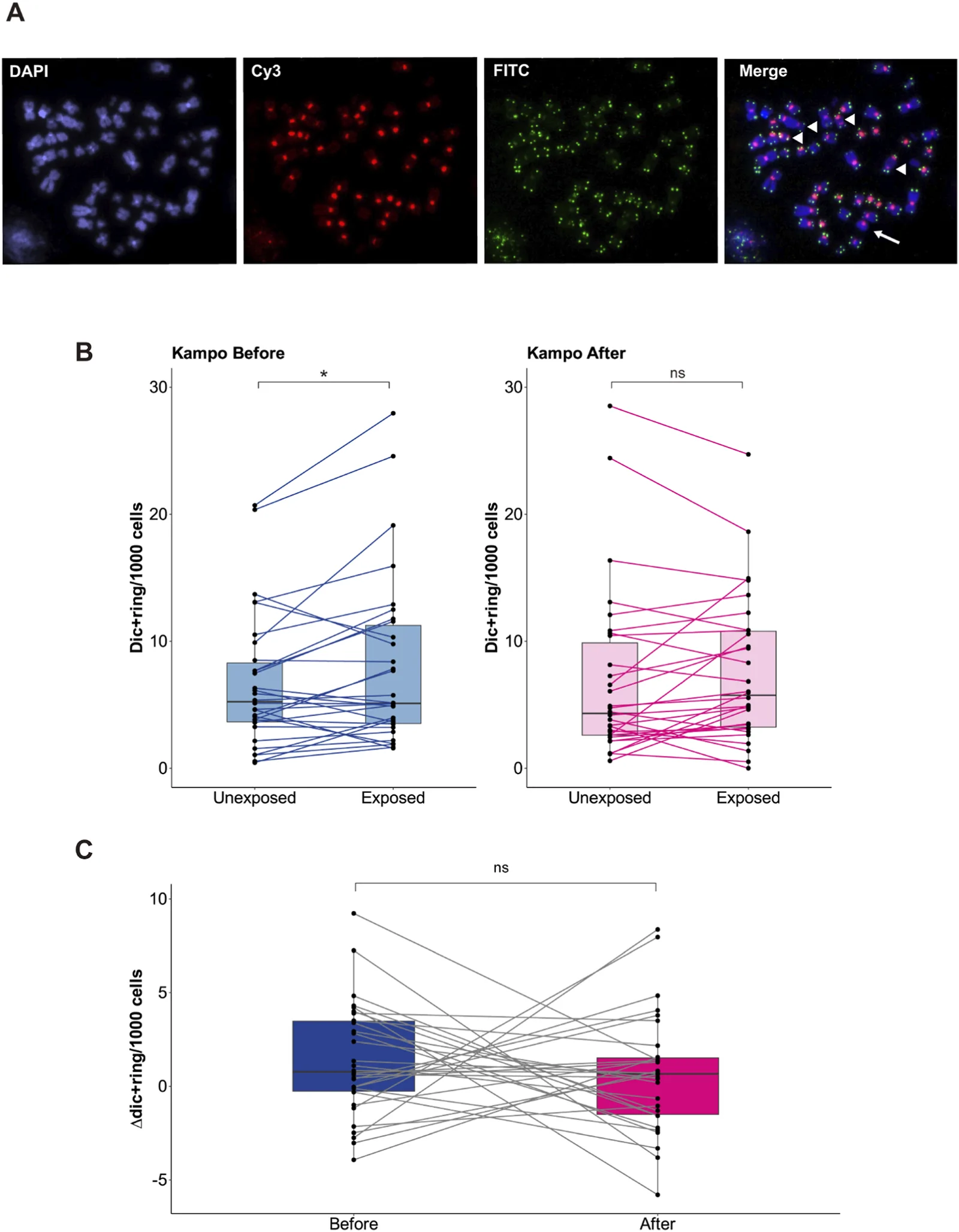
*γ*-H2AX focus formation induced by a CT scan before and after Kampo treatment. **(A)** Representative images of γ-H2AX foci in a PBL. DAPI-stained nuclear DNA (blue), Cy3-labeld *γ*-H2AX foci (red) and the merged image are shown. Scale bar: 2 µm. **(B)** Changes in number of *γ*-H2AX foci by CT exposure before (left) and after (right) Kampo treatment (before; p = 0.023, after; p = 0.428). **(C)** Comparison of CT-induced increase in number of *γ*-H2AX foci (exposed–unexposed) before and after Kampo treatment (p = 0.428). A total of 30 patients treated with bukuryoin, rikkunshito, heiisan or nichinto were included. Wilcoxon matched-pairs signed-rank test was used to compare each group. *p < 0.05, ***p < 0.001, ns: not significant.

γ-H2AX foci were measured as a sensitive marker of early DNA double-strand breaks (Figure 4A). A strong, significant difference was observed before Kampo treatment (Δ = 0.18, IQR, 0.06 to 0.29, p = 2.97 × 10^−4^; Figure 4B left; Supplementary Table S4). After Kampo treatment, this difference was attenuated (Δ = 0.09, IQR, −0.07 to 0.08, p = 1.89 × 10^−2^; Figure 4B right; Supplementary Table S5), consistent with a radioprotective effect. However, the pre-post change in the between-group difference was not statistically significant (Δ = −0.12, IQR, −0.21 to −0.08, p = 0.214; Figure 4C; Table 6). The CT × Kampo interaction in the LMM was negative (β = −0.09) but not statistically significant (95% CI: −2.41 to 0.07, p = 0.292) (Table 7).

**TABLE 6:** Comparison of CT exposure-related changes in number of *γ*-H2AX foci before and after Kampo treatment.

Group	n	Median (IQR)	Mean ± SD
All patients	33	−0.12 (−0.21, −0.08)	0.17 ± 0.42
Kampo formula subgroup			
Bukuryoin	15	−0.10 (−5.05, −0.17)	−1.61 ± 5.49
Rikkunshito	6	−0.10 (−0.86, −0.22)	0.15 ± 0.76
Heiisan	11	−0.17 (−0.21, −0.09)	−0.05 ± 0.19
Component subgroup			
Citri unshiu pericarpium			
≧3 g	27	−0.12 (−0.21, −0.09)	−0.05 ± 0.32
<3 g	5	−0.10 (−0.86, −0.22)	0.15 ± 0.76

**TABLE 7:** Comparison of fitted data of LMM model and Wilcoxon matched pair singed rank test of γ-H2AX data.

Group	n	LMM	​	Wilcoxon signed-rank
		Kampo × exposure inter-action effect (95% CI)	p-value	p-value
All patients	33	−0.085 (−2.410, 0.071)	0.292	0.214
Kampo formula subgroup				
Bukuryoin	15	−0.023 (−0.225, 0.180)	0.830	0.847
Rikkunshito	6	−0.222 (−0.719, 0.276)	0.413	0.528
Heiisan	11	−0.092 (−0.364, 0.180)	0.521	0.054
Component subgroup				
Citri unshiu pericarpium				
≧3 g	25	−0.054 (−0.212, 0.103)	0.503	0.269
<3 g	6	−0.222 (−0.719, 0.276)	0.413	0.528

In the subgroup analysis, neither bukuryoin nor rikkunshito showed evidence of radioprotective effects (Supplementary Figures S3A-D). For heiisan treatment, the pre-treatment difference in CAs was significant (Δ = 0.23, IQR, 0.12 to 0.30, p = 6.84 × 10^−3^; Supplementary Figure S3E). After heiisan treatment, the difference was attenuated and not significant (Δ = 0.09, IQR, 0.02 to 0.20, p = 6.74 × 10^−2^; Supplementary Figure S3E). Despite this, the pre-post change in the between-group difference was not statistically significant (median Δ = −0.17, IQR, −0.21 to −0.09; p = 5.37 × 10^−2^; Table 6). The CT × Kampo interaction in the LMM was negative (β = −0.09) but not significant (95% CI: −0.36 to 0.18, p = 0.521) (Table 7). Component-based subgroup analysis also showed no statistically significant results, although trends were consistent with the overall protective effects. The lower ICC (0.65) for γ-H2AX may have contributed to reduced LMM sensitivity. These findings align with the attenuating effects observed in the CA analysis.

### 3.5 Sample size estimation for future definitive trial

Using the prespecified parameters (two sided α = 0.05, power = 0.80), we based the power calculation on CAs because a higher ICC denotes lower within assay error variance (ICC = 0.89 vs. 0.65 for γ H2AX). We then estimated the sample size for a future definitive trial using two approaches (Table 8).

**TABLE 8:** Sample size calculation using two distinct statistical analyses using PNA-FISH data.

Items	LMM	Wilcoxon signed-rank
Significance level *α* (two-sided)	0.05	0.05
Power (1-*β*)	0.8	0.8
Estimated group differences	−0.72	−0.72
SD calculation	Estimated from ICC^a^	Pooled SD^b^
SD	3.0	3.1
Effect size (Cohen’s d)	−0.25	−0.23
Sample size (single arm)	274	292

^a^ SD, estimated from ICC, and residual variance from the LMM.

^b^ Pooled SD, was calculated from both groups.

SD, standard deviation; ICC, intraclass correlation coefficient.

These calculations were intended to inform the design of a future parallel-group randomized controlled trial and therefore were based on an unpaired comparison framework.

In the LMM based approach, targeting the Kampo × CT interaction and using the ICC derived SD of 3.0, the anticipated contrast of −0.72 units corresponded to Cohen’s d = −0.25 and required 274 evaluable patients per arm (548 total). As a non parametric sensitivity analysis using Wilcoxon signed rank test and the pooled SD of 3.1, the same contrast yielded Cohen’s d = −0.23 and 292 evaluable patients per arm (584 total). The negative sign reflects an expected reduction in the outcome; magnitude is interpreted using |d| (≈0.2 small, 0.5 medium, 0.8 large). These figures represent evaluable patients and should be inflated to account for attrition and screening to randomization yield when setting recruitment targets. Overall, both methods converge on a planning range of approximately 275–295 evaluable patients per arm prior to inflation for retention.

## 4 Discussion

This exploratory study suggests that Kampo formulas containing D-limonene may mitigate CT induced genotoxicity in human lymphocytes. Before treatment, low dose CT produced a measurable rise in CAs, consistent with prior reports of CT related biological effects (Shi et al., 2018; Sakane et al., 2020) and large cohort data linking medical CT in youth to later cancer risk (Hauptmann et al., 2023; Smith-Bindman et al., 2025; Pearce et al., 2012; Mathews et al., 2013). After Kampo treatment, the unexposed–exposed difference in CAs was attenuated, and the difference-in-differences indicated a non-significant reduction; γ-H2AX showed a similar qualitative pattern. Overall, these findings are compatible with a modest radioprotective effect that warrants confirmation in a definitive trial.

The biological plausibility is supported by *in vitro* data showing ALDH3A1 activation across formulas (Supplementary Figure S1) and prior work demonstrating that enhancing ALDH activity limits radiation injury by detoxifying reactive aldehydes from lipid peroxidation (Saiki et al., 2018; Ning et al., 2012). These aldehyde scavenging pathways provide a coherent mechanism for the observed attenuation. While D limonene is likely a key contributor, the multi-metabolite nature of Kampo formulas suggests potential synergism, meriting further mechanistic study.

Methodologically, our results highlight the importance of endpoint selection and measurement reliability. PNA FISH exhibited high reproducibility (ICC = 0.89) and captures cumulative chromosomal damage of recognized clinical relevance (Bonassi et al., 2008), whereas γ H2AX (ICC = 0.65) reflects early, transient DNA double strand break signaling and is sensitive to sampling and inter individual variability (Löbrich et al., 2010). Cytogenetic assays are considered robust at low doses when rigorously performed (International Atomic Energy Agency (IAEA), 2011). On this basis, PNA FISH is the preferable primary endpoint for a confirmatory trial, with γ H2AX as a mechanistic secondary outcome.

Subgroup findings should be interpreted cautiously but help refine hypotheses. The bukuryoin subgroup mirrored the overall attenuation in CAs, whereas rikkunshito and heiisan did not. For γ H2AX, heiisan showed a borderline decrease by paired test (p = 0.054) that was not confirmed in the mixed effects model. Given that each formula contains multiple bioactive metabolites with antioxidant and gastrointestinal effects (Riedlinger et al., 2001; Shin et al., 2011), the complexity of multiple metabolites rather than any single metabolite likely explains heterogeneity in response. Furthermore, γ-H2AX represents an early and transient marker of DNA double-strand break signaling, whereas PNA-FISH captures accumulated chromosomal damage. These biological and methodological differences may partly explain the discrepancy between the PNA-FISH and γ-H2AX findings.

The potential contribution of D-limonene to the observed radioprotective effects warrants further consideration. In our exploratory subgroup analysis, patients receiving Kampo formulas containing ≥3.0 g/day of Citri Unshiu Pericarpium exhibited a larger negative CT × Kampo interaction estimate than those receiving lower amounts, although statistical significance was not achieved. While this finding should be interpreted cautiously because of the limited sample size and the indirect use of Citri Unshiu Pericarpium dose as a surrogate for D-limonene exposure, it is consistent with a possible contribution of higher D-limonene exposure to the observed effects. A biologically plausible explanation is that D-limonene may enhance ALDH3A1-mediated detoxification of radiation-induced reactive aldehydes, thereby reducing oxidative and cytogenetic damage. Previous studies have identified D-limonene as an activator of ALDH3A1 and demonstrated its protective effects against radiation-induced tissue injury (Saiki et al., 2018). Consistent with this mechanism, all Kampo formulas tested in the present study activated recombinant human ALDH3A1 *in vitro*. However, D-limonene content was not directly quantified, ALDH3A1 activity was not measured in patient lymphocytes or blood samples, and Citri Unshiu Pericarpium contains multiple bioactive metabolites besides D-limonene. Therefore, the present findings should be regarded as exploratory, and future studies incorporating direct quantification of D-limonene exposure and longitudinal assessment of ALDH activity will be necessary to clarify the contribution of D-limonene and the underlying mechanisms.

Dose considerations are important. Pharmacokinetic data indicate that achieving pharmacologically active plasma concentrations of D limonene in humans requires approximately ∼2 g/day for 2 weeks (Ning et al., 2012), whereas our formulas deliver milligram range amounts within multi herbal regimens. Accordingly, the observed attenuation is consistent with mitigation of low dose CT effects and may reflect synergistic actions of multiple constituents. Since this study focused on low dose CT exposure, these findings should not be extrapolated to high dose irradiation.

This study has several limitations. First, the clinical component was conducted without a randomized control group, which limits the ability to fully exclude potential confounding factors and temporal variations unrelated to Kampo treatment. Second, the sample size was modest and the subgroup distribution was unbalanced, particularly for nichinto-treated patients, which may have reduced statistical power and limited the generalizability of the findings. Third, the treatment period was relatively short (approximately 19 days), whereas longer durations of Kampo administration are commonly used in clinical practice and may produce different biological effects. In addition, the irradiation experiments were performed using an *in vitro* blood irradiation model with a human body phantom. Although this approach was designed to simulate CT exposure conditions, it cannot fully reproduce the complex biological responses that occur during *in vivo* radiation exposure. Another limitation is the discrepancy between the estimated human exposure levels of D-limonene, which are generally in the milligram range following Kampo administration, and the substantially higher doses frequently used in experimental animal studies. Therefore, caution is warranted when directly comparing mechanistic findings across different experimental systems. Finally, although our results suggest that ALDH activity may contribute to the protective effects of D-limonene against CT-associated chromosomal damage, the proposed mechanism has not yet been directly validated *in vivo* and should therefore be regarded as preliminary. To obtain more definitive evidence, we propose a parallel-group randomized controlled trial powered on PNA-FISH (ICC = 0.89), which, based on our estimates, would require approximately 275–295 evaluable participants per arm before accounting for attrition, with γ-H2AX included as a prespecified secondary endpoint. Such a study would help determine whether D-limonene-containing Kampo formulas meaningfully mitigate CT-associated genotoxicity in humans and would provide an opportunity to further investigate the underlying biological mechanisms.

## 5 Conclusion

In this single arm pilot study, we explored the radioprotective potential of D-limonene-containing Kampo formulas against low-dose CT-induced genotoxicity in human lymphocytes. Of the two biomarkers assessed, CAs measured by PNA FISH demonstrated higher reliability than γ H2AX, as reflected by a superior ICC. Kampo formulas showed a consistent trend toward radioprotection, although the effects did not reach statistical significance. These findings suggest a possible radioprotective effect of D-limonene-containing Kampo formulations and support the use of PNA-FISH as a potentially useful endpoint for future trials. While no definitive conclusions can be drawn from this preliminary study, the observed trends may warrant further investigation in adequately powered randomized controlled trials.

## Data Availability

The original contributions presented in the study are included in the article/Supplementary Material, further inquiries can be directed to the corresponding authors.

## Supplementary material

Supplementary figure S1 Activation of human recombinant ALDH3A1 isozyme by D-limonene-containing Kampo formulas. Bukuryoin, rikkunshito, heiisan and nichinto showed activation of ALDH3A1 enzyme activity of 217%, 200%, 244% and 183% over DMSO control (100%). Percent of enzyme activity is based on comparison with assays using DMSO as the control. Data are presented as means ± SD, n = 3, *P < 0.05, **P < 0.01.

SUPPLEMENTARY FIGURE S2 Subgroup analysis of CAs induced by CT scans before and after Kampo treatment. **(A)** Changes in CAs by CT exposure before (left) and after (right) bukuryoin (befoe; p = 0.040, after; p = 0.735), **(C)** rikkunshito (before; p = 0.313, after; p = 0.625), **(E)** heiisan (before; p = 0.102, after; p = 0.898). **(B)** Comparison of CT-induced increase in CAs (exposed – unexposed) before and after bukuryoin (p = 0.244), **(D)** rikkunshito (p = 0.438), **(F)** heiisan (p = 0.413). Wilcoxon matched-pairs signed-rank test was used to compare each group. *p < 0.05; ns, not significant.

SUPPLEMENTARY FIGURE S3 Subgroup analysis of *γ*-H2AX foci induced by CT scans before and after Kampo treatment. **(A)** Changes in CAs by CT exposure before (left) and after (right) bukuryoin (before; p = 1.70 × 10^−2^, after; p = 4.46 × 10^−2^), **(C)** rikkunshito (before; p = 0.313, after; p = 0.844), **(E)** heiisan (before; p = 6.84 × 10^−3^, after; p = 6.74 × 10^−2^). **(B)** Comparison of CT-induced increase in CAs (exposed – unexposed) before and after bukuryoin (p = 0.847), **(D)** rikkunshito (p = 0.528), **(F)** heiisan (p = 5.37 × 10^−2^). Wilcoxon matched-pairs signed-rank test was used to compare each group. *p < 0.05; †p < 0.1; ns, not significant.
