# Supplementary material for: Exploratory assessment of the potential protective effects of Kampo treatment against low dose medical radiation: a single arm prospective observational study
Source: Front Pharmacol. 2026 Aug 7;17:1888108. doi: 10.3389/fphar.2026.1888108 (PMC13493526; doi:10.3389/fphar.2026.1888108)
Supplement: Supplementary file 1 [file Supplementaryfile2.pdf]

Revised: May 2020 (8th version)

Standard Commodity Classification No. of Japan
875200

■43■

- Kampo-preparation-

TSUMURA Rikkunshito Extract Granules for Ethical Use

Storage
Store in light-resistant, air-tight containers.

Expiration date
Use before the expiration date indicated on the container and the outer package.

Approval No.	(61AM)3260
Date of listing in the NHI reimbursement price	October 1986
Date of initial marketing in Japan	October 1986
Date of latest reevaluation	April 2014

DESCRIPTION

Composition	7.5 g of TSUMURA Rikkunshito extract granules (hereafter TJ-43) contains 4.0 g of a dried extract of the following mixed crude drugs.<br>JP Atractylodes Lancea Rhizome ...... 4.0 g<br>JP Ginseng .... 4.0 g<br>JP Pinellia Tuber .... 4.0 g<br>JP Poria Sclerotium .... 4.0 g<br>JP Jujube .... 2.0 g<br>JP Citrus Unshiu Peel .... 2.0 g<br>JP Glycyrrhiza .... 1.0 g<br>JP Ginger .... 0.5 g<br>(JP: The Japanese Pharmacopoeia)	
	Inactive ingredients	JP Magnesium Stearate<br>JP Lactose Hydrate<br>Sucrose Esters of Fatty Acids
Description	Dosage form	Granules
	Color	Light grayish-brown
	Smell	Characteristic smell
	Taste	Sweet
	ID code	TSUMURA/43

INDICATIONS

TJ-43 is indicated for the relief of the following symptoms of those patients with weak stomach, loss of appetite and full stomach pit, and those who are easily fatigued, anemic and likely to have cold limbs:

Gastritis, gastric atony, gastroptosis, maldigestion, anorexia, gastric pain, vomiting.

DOSAGE AND ADMINISTRATION

The usual adult dose is 7.5 g/day orally in 2 or 3 divided doses before or between meals. The dosage may be adjusted according to the patient's age and body weight, and symptoms.

PRECAUTIONS

1. Important Precautions

(1) When TJ-43 is used, the patient's "SHO" (constitution/symptoms) should be taken into account. The patient's progress should be carefully monitored, and if no improvement in symptoms/findings is observed, continuous treatment should be avoided.

(2) Since TJ-43 contains Glycyrrhiza, careful attention should be paid to the serum potassium level, blood pressure, etc., and if any abnormality is observed, administration should be discontinued.

(3) When TJ-43 is coadministered with other Kampo-preparations (Japanese traditional herbal medicines), etc., attention should be paid to the duplication of the contained crude drugs.

SHO: The term "SHO" refers to a particular pathological status of a patient evaluated by the Kampo diagnosis, and is patterned according to the patient's constitution, symptoms, etc. Kampo-preparations (Japanese traditional herbal medicines) should be used after confirmation that it is suitable for the identified "SHO" of the patient.

2. Drug Interactions

Precautions for coadministration (TJ-43 should be administered with care when coadministered with the following drugs.)

Drugs	Signs, Symptoms, and Treatment	Mechanism and Risk Factors
(1) Preparations containing Glycyrrhiza<br>(2) Preparations containing glycyrrhizinic acid or glycyrrhizinates	Pseudoaldosteronism is likely to occur. Besides, myopathy is likely to occur as a result of hypokalemia.<br>(Refer to the section "Clinically significant adverse reactions".)	Since glycyrrhizinic acid has an accelerating action on the potassium excretion at the renal tubules, an acceleration of decrease in the serum potassium level has been suggested.

3. Adverse Reactions

The incidence of adverse reactions in a clinical survey of adverse reaction.[1)]

(1) Clinically significant adverse reactions

1) **Pseudoaldosteronism**(incidence unknown): Pseudoaldosteronism such as hypokalemia, increased blood pressure, retention of sodium/body fluid, edema, increased body weight, etc. may occur. The patient should be carefully monitored (measurement of serum potassium level, etc.), and if any abnormality is observed, administration should be discontinued and appropriate measures such as administration of potassium preparations should be taken.

2) **Myopathy**(incidence unknown): Myopathy may occur as a result of hypokalemia. The patient should be carefully monitored, and if any abnormality such as weakness, convulsion/paralysis of limbs, etc. are observed, administration should be discontinued and appropriate measures such as administration of potassium preparations should be taken.

3) **Hepatic dysfunction and jaundice**(incidence unknown): Hepatic dysfunction and/or jaundice with remarkable elevation of AST, ALT, Al-P and γ-GTP etc. may occur. The patient should be carefully monitored for abnormal findings. Administration should be discontinued and appropriate therapeutic measures should be taken, if abnormalities are observed.

(2) Other adverse reactions

	5%> ≥0.1%	<0.1%
**Hypersensitivity** Note 1)		Rash, Urticaria, etc.
**Hepatic**	Abnormality of hepatic function [containing increased AST, ALT, Al-P and γ-GTP levels, etc.]	
**Gastrointestinal**	Nausea	Feeling of enlarged abdomen, Diarrhea, etc.
**Others**	Hypokalemia, High blood pressure [containing increased blood pressure], Edema	

Note 1) If such symptoms are observed, administration should be discontinued.

4. Use in the Elderly

Because elderly patients often have reduced physiological function, careful supervision and measures such as reducing the dose are recommended.

5. Use during Pregnancy, Delivery or Lactation

The safety of TJ-43 in pregnant women has not been established. Therefore, TJ-43 should be used in pregnant women, women who may possibly be pregnant only if the expected therapeutic benefits outweigh the possible risks associated with treatment.

6. Pediatric Use

The safety of TJ-43 in children has not been established. [Insufficient clinical data.]

PHARMACOLOGY

1. Enhancement of digestive motility

(1)Administration of TJ-43 for the patients (n=7) suffering from chronic gastritis with delayed gastric emptying (experiencing anorexia etc.) improved the ability of gastric emptying at 2 and 4 weeks post-administration (acetaminophen test)[2)]

(2) Oral administration of Rikkunshito shortened the cycle and total elapsed time (TET) of interdigestive migrating contractions (IMC) in dogs[3)].

2. Actions on gastric adaptive relaxation

Rikkunshito enhanced internal pressure-dependent gastric adaptive relaxation in the isolated guinea pig stomach under a cholinergic and adrenergic blocking condition (in vitro)[4)].

3. Effect on gastric mucosal injury

(1) Oral administration of Rikkunshito suppressed the development of gastric mucosal lesions induced by compound 48/80 in rats[5)].

(2) Oral pretreatment with Rikkunshito suppressed development of gastric mucosal lesions induced by indomethacin[6)] or repeated electrical stimulation[7)] and suppressed parietal cell injury of the gastric mucosa induced by adriamycin[8)] in rats.

4. Suppression of decrease in gastric mucosal blood flow

Oral pretreatment with Rikkunshito suppressed the decrease in gastric mucosal blood flow induced by repetitive electrical stimulation of the gastric artery in rats[7)].

5. Stimulation of appetite

Oral administration of Rikkunshito suppressed the decrease in food intake in a stress-model mouse induced by environmental change[9)], anorexia-model rats induced by cisplatin[10)], and aging mice[11)].

6. Mechanism of action

Rikkunshito shows pharmacological effects via the following actions:

(1) Actions on gastric adaptive relaxation

Enhanced internal pressure-dependent gastric adaptive relaxation in the isolated guinea pig stomach was disappeared by NO synthase inhibitor, NG-nitro L-arginine, and reappeared by the addition of Rikkunshito (in vitro)[4)].

(2) Effect on gastric mucosal injury

- Oral pretreatment with Rikkunshito suppressed the elevation of myeloperoxidase (MPO) activity induced by indomethacin[6)] or by repetitive electrical stimulation of the gastric artery[7)] in rats.
- Oral pretreatment with Rikkunshito suppressed leukocyte infiltration in the lower part of the fundic gland induced by indomethacin[6)], and also suppressed the increase in PAF formation and decrease in leu-

kocyte count in the gastric mucosa induced by repeated electrical stimulation of the gastric artery[7] in rats.

- Oral administration of Rikkunshito suppressed the increase in lipid peroxide concentration, reduction of Se-containing glutathione peroxidase activity, and elevation of MPO activity in the gastric mucosa induced by compound 48/80 in rats[5].
- Oral pretreatment of rats with Rikkunshito suppressed the ethanol-induced decrease of mucus volume in the deep mucosa of the gastric corpus therefor increased mucus volume at gastric surface layer[12].

(3) Scavenging of reactive oxygen species

Rikkunshito exerted a scavenging effect on superoxide anion and hydroxyl radical and inhibitory effect on MPO activity in rat gastric mucosa *in vitro*[5].

(4) Stimulation of appetite

- Oral administration of Rikkunshito improved the decrease in food intake, decrease in gastrointestinal motility, and delayed gastric emptying in rats with gastrointestinal dysfunction induced by SSRI through indirect stimulation of ghrelin secretion via 5-HT_{2C} receptor antagonism[13].
- Oral administration of Rikkunshito improved the decrease in blood concentration of ghrelin and suppressed the decrease in food intake in a rat model of anorexia induced by cisplatin. The improving effect on food intake was abolished by the concomitant administration of the ghrelin receptor antagonist [D-Lys^3]-GHRP- 6[10].

PACKAGING

Bottles of 500 g and boxes of 5 kg (500 g × 10 bottles)
2.5 g × 42 packets
2.5 g × 189 packets

REFERENCES

1) Suzuki, Y. et al. Prog. Med. 2020, 40(3), p.305
2) Harasawa, S. et al. Gastroenterology. 1990, 12(2), p.215.
3) Murakuni, H. et al. Jpn. J. Oriental Medicine. 1992, 43(2), p.255.
4) Hayakawa, T. Et al. Drugs Exp. Clin. Res. 1999, 25(5), p.211.
5) Kobayashi, T. et al. J. Traditional Med. 1994, 11(2), p.123.
6) Murakami, K. Jpn. J. Oriental Medicine. 1997, 48(1), p.1.
7) Kurose, I. et al. Pathophysiology. 1995, 2, p.153.
8) Yu, X. M. et al. Acta Histochem. Cytochem. 1995, 28(6), p.539.
9) Saegusa, Y. et al. Am. J. Physiol. Endocrinol. Metab. 2011, 301(4), p.685.
10) Takeda, H. et al. Gastroenterology. 2008, 134(7), p.2004.
11) Takeda, H. et al. Endocrinology. 2010, 151(1), p.244.
12) Ogata, Y. et al. Diagnosis and Treatment. 1992, 80(7), p.1257.
13) Fujitsuka, N. et al. Biol. Psychiatry. 2009, 65(9), p.748.

REQUEST FOR LITERATURE SHOULD BE MADE TO:

Consumer Information Services Center
Tsumura & Co.
2-17-11 Akasaka, Minato-ku, Tokyo 107-8521, Japan
TEL: 0120-329970 FAX: 03-5574-6610

Manufactured and Distributed by:

Tsumura & Co.
2-17-11 Akasaka, Minato-ku, Tokyo 107-8521, Japan

Rikkunshito

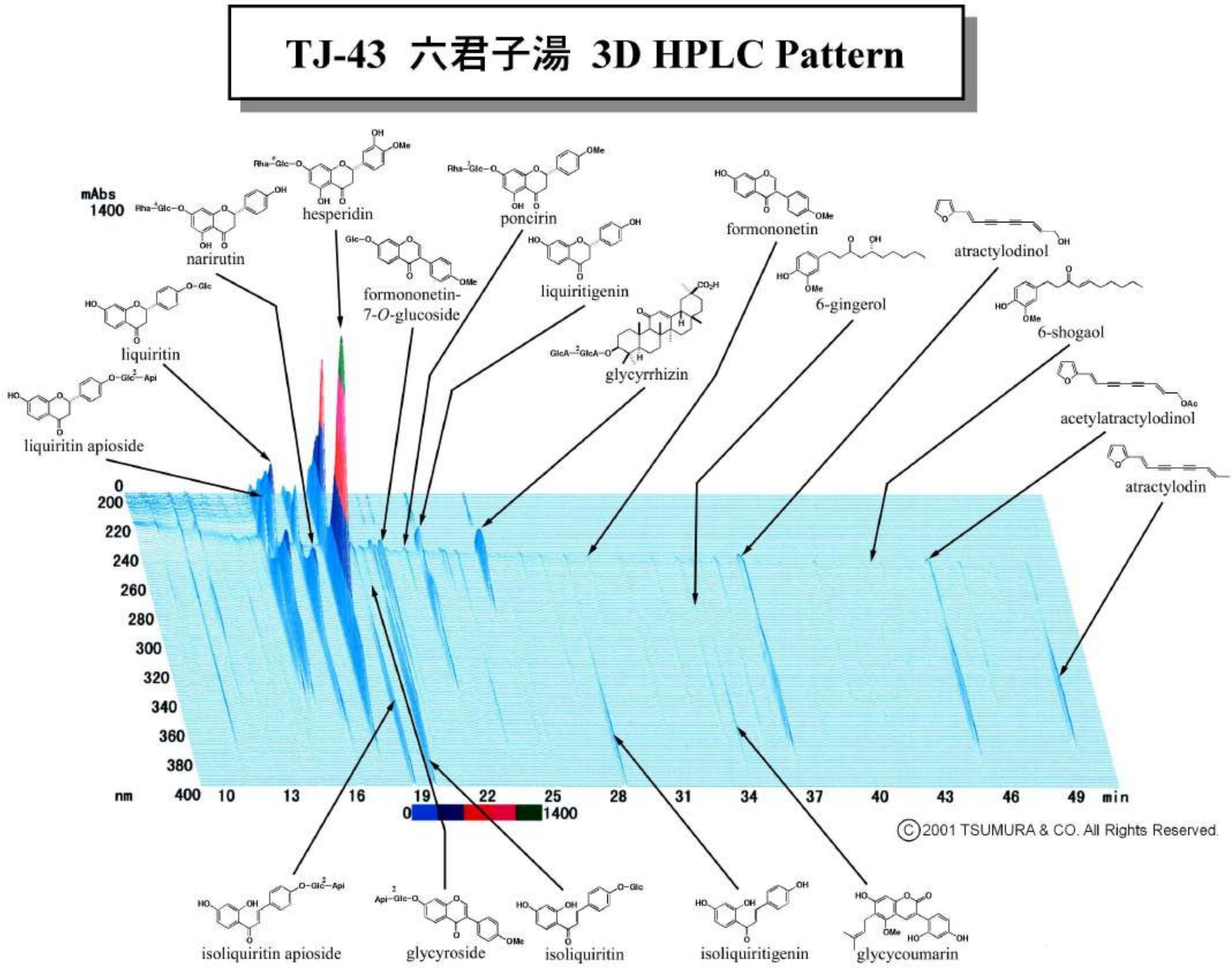

©2001 TSUMURA & CO. All Rights Reserved.

Revised: March 2013 (5th version)

Standard Commodity Classification No. of Japan
875200

- Kampo-preparation-

TSUMURA Bukuryoin Extract Granules for Ethical Use

<bukuryoin>

Storage
Store in light-resistant, air-tight containers.

Approval No.	(61AM)1130
Date of listing in the NHI reimbursement price	October 1986
Date of initial marketing in Japan	October 1986

Expiration date
Use before the expiration date indicated on the container and the outer package.

DESCRIPTION

Composition	7.5 g of TSUMURA Bukuryoin extract granules contains 2.75 g of a dried extract of the following mixed crude drugs.<br>JP Poria Sclerotium .... 5.0 g<br>JP Atractylodes Lancea Rhizome …… 4.0 g<br>JP Citrus Unshiu Peel .... 3.0 g<br>JP Ginseng .... 3.0 g<br>JP Immature Orange .... 1.5 g<br>JP Ginger .... 1.0 g<br>(JP: The Japanese Pharmacopoeia)	
	Inactive ingredients	JP Magnesium Stearate<br>JP Lactose Hydrate
Description	Dosage form	Granules
	Color	Light grayish-brown
	Smell	Characteristic smell
	Taste	Bitter and pungent
	ID code	TSUMURA/69

INDICATIONS

TSUMURA Bukuryoin Extract Granules (hereafter TJ-69) is indicated for the relief of the following symptoms of those patients with nausea or heartburn and decreased urine volume:
Gastritis, gastric atony, and excessive fluid retention in the stomach

DOSAGE AND ADMINISTRATION

The usual adult dose is 7.5 g/day orally in 2 or 3 divided doses before or between meals. The dosage may be adjusted according to the patient's age and body weight, and symptoms.

PRECAUTIONS

1. Important Precautions

(1) When TJ-69 is used, the patient’s “SHO” (constitution/symptoms) should be taken into account. The patient’s progress should be carefully monitored, and if no improvement in symptoms/findings is observed, continuous treatment should be avoided.

(2) When TJ-69 is coadministered with other Kampo-preparations (Japanese traditional herbal medicines), etc., attention should be paid to the duplication of the contained crude drugs.

SHO: The term “SHO” refers to a particular pathological status of a patient evaluated by the Kampo diagnosis, and is patterned according to the patient’s constitution, symptoms, etc. Kampo-preparations (Japanese traditional herbal medicines) should be used after confirmation that it is suitable for the identified “SHO” of the patient.

2. Adverse Reactions

TJ-69 has not been investigated (drug use investigations, etc.) to determine the incidence of adverse reactions. Therefore, the incidence of adverse reactions is not known.

	Incidence unknown
**Hypersensitivity** Note 1)	Rash, Urticaria, etc.

Note 1) If such symptoms are observed, administration should be discontinued.

3. Use in the Elderly

Because elderly patients often have reduced physiological function, careful supervision and measures such as reducing the dose are recommended.

4. Use during Pregnancy, Delivery or Lactation

The safety of TJ-69 in pregnant women has not been established. Therefore, TJ-69 should be used in pregnant women, women who may possibly be pregnant only if the expected therapeutic benefits outweigh the possible risks associated with treatment.

5. Pediatric Use

The safety of TJ-69 in children has not been established. [Insufficient clinical data]

PACKAGING

Bottles of 500 g
2.5 g × 42 packets
2.5 g × 189 packets

REQUEST FOR LITERATURE SHOULD BE MADE TO:

Consumer Information Services Center
Tsumura & Co.
2-17-11 Akasaka, Minato-ku, Tokyo 107-8521, Japan

Manufactured and Distributed by:

Tsumura & Co.
2-17-11 Akasaka, Minato-ku, Tokyo 107-8521, Japan

Bukuryoin

TJ-69 茯苓飲 3D HPLC Pattern

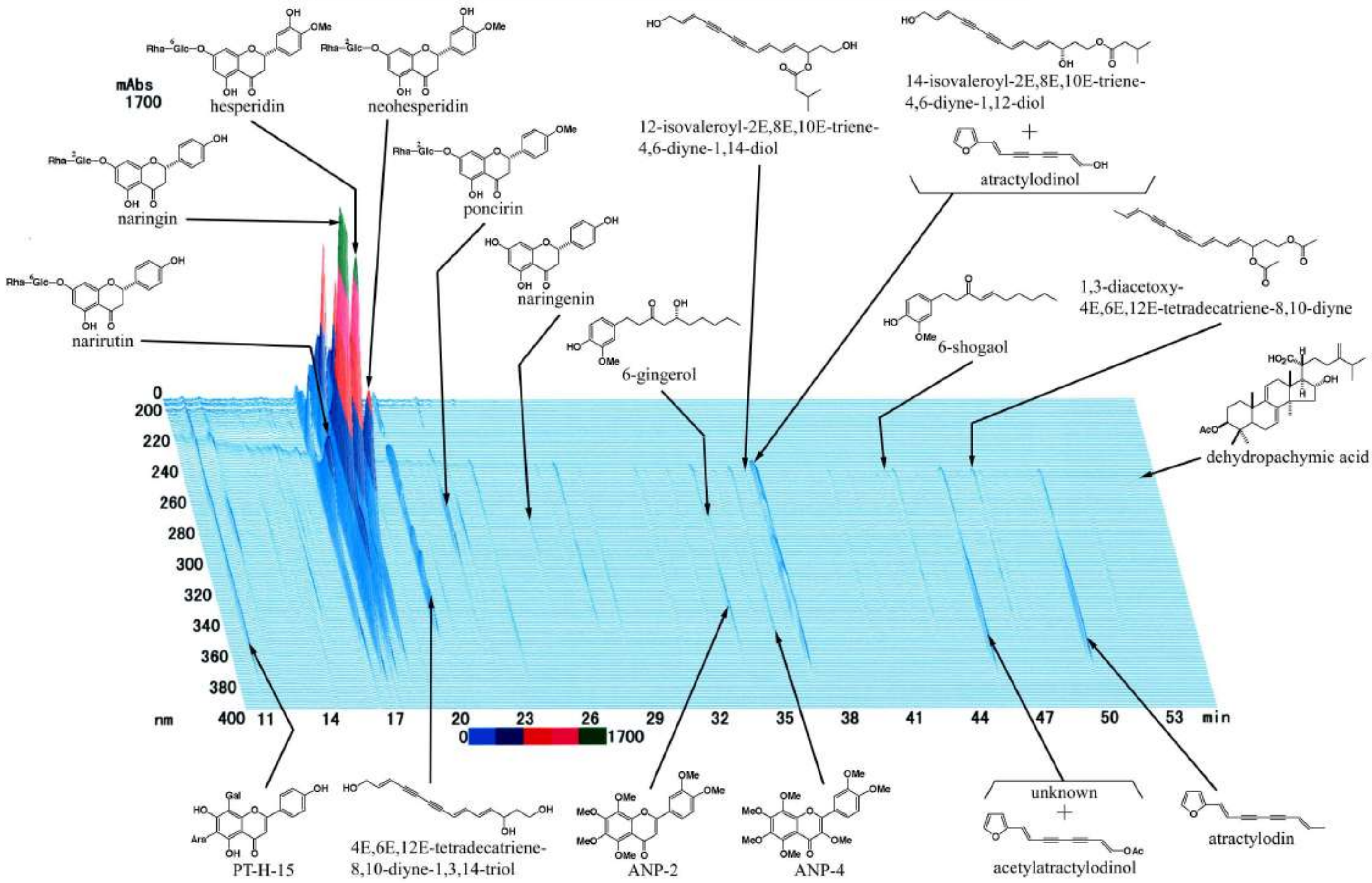

©2001 TSUMURA & CO. All Rights Reserved.

Revised: March 2013 (5th version)

Standard Commodity Classification No. of Japan
875200

- Kampo-preparation-

TSUMURA Heiisan Extract Granules for Ethical Use

<heiisan>

Storage
Store in light-resistant, air-tight containers.

Approval No.	(61AM)3273
Date of listing in the NHI reimbursement price	October 1986
Date of initial marketing in Japan	October 1986

Expiration date
Use before the expiration date indicated on the container and the outer package.

DESCRIPTION

Composition	7.5 g of TSUMURA Heiisan extract granules contains 3.25 g of a dried extract of the following mixed crude drugs.<br>JP Atractylodes Lancea Rhizome .... 4.0 g<br>JP Magnolia Bark ……………………. 3.0 g<br>JP Citrus Unshiu Peel .... 3.0 g<br>JP Jujube .... 2.0 g<br>JP Glycyrrhiza .... 1.0 g<br>JP Ginger .... 0.5 g<br>(JP: The Japanese Pharmacopoeia)	
	Inactive ingredients	JP Light Anhydrous Silicic Acid<br>JP Magnesium Stearate<br>JP Lactose Hydrate
Description	Dosage form	Granules
	Color	Light brown
	Smell	Characteristic smell
	Taste	Sweet and slightly acrid
	ID code	TSUMURA/79

INDICATIONS

TSUMURA Heiisan Extract Granules (hereafter TJ-79) is indicated for the relief of the following symptoms of those patients with a heavy stomach feeling and a tendency to dyspepsia:

Acute or chronic esogastritis, gastric atony, dyspepsia, and anorexia

DOSAGE AND ADMINISTRATION

The usual adult dose is 7.5 g/day orally in 2 or 3 divided doses before or between meals. The dosage may be adjusted according to the patient's age and body weight, and symptoms.

PRECAUTIONS

1. Important Precautions

(1) When TJ-79 is used, the patient's "SHO" (constitution/symptoms) should be taken into account. The patient's progress should be carefully monitored, and if no improvement in symptoms/findings is observed, continuous treatment should be avoided.

(2) Since TJ-79 contains Glycyrrhiza, careful attention should be paid to the serum potassium level, blood pressure, etc., and if any abnormality is observed, administration should be discontinued.

(3) When TJ-79 is coadministered with other Kampo-preparations (Japanese traditional herbal medicines), etc., attention should be paid to the duplication of the contained crude drugs.

SHO: The term "SHO" refers to a particular pathological status of a patient evaluated by the Kampo diagnosis, and is patterned according to the patient's constitution, symptoms, etc. Kampo-preparations (Japanese traditional herbal medicines) should be used after confirmation that it is suitable for the identified "SHO" of the patient.

2. Drug Interactions

Precautions for coadministration (TJ-79 should be administered with care when coadministered with the following drugs.)

Drugs	Signs, Symptoms, and Treatment	Mechanism and Risk Factors
(1) Preparations containing Glycyrrhiza<br>(2) Preparations containing glycyrrhizinic acid or glycyrrhizinates	Pseudoaldosteronism is likely to occur. Besides, myopathy is likely to occur as a result of hypokalemia.<br>(Refer to the section "Clinically significant adverse reactions".)	Since glycyrrhizinic acid has an accelerating action on the potassium excretion at the renal tubules, an acceleration of decrease in the serum potassium level has been suggested.

3. Adverse Reactions

TJ-79 has not been investigated (drug use investigations, etc.) to determine the incidence of adverse reactions. Therefore, the incidence of adverse reactions is not known.

Clinically significant adverse reactions

(1) Pseudoaldosteronism: Pseudoaldosteronism such as hypokalemia, increased blood pressure, retention of sodium/body fluid, edema, increased body weight, etc. may occur. The patient should be carefully monitored (measurement of serum potassium level, etc.), and if any abnormality is observed, administration should be discontinued and appropriate measures such as administration of potassium preparations should be taken.

(2) Myopathy: Myopathy may occur as a result of hypokalemia. The patient should be carefully monitored, and if any abnormality such as weakness, convulsion/paralysis of limbs, etc. are observed, administration should be discontinued and appropriate measures such as administration of potassium preparations should be taken.

4. Use in the Elderly

Because elderly patients often have reduced physiological function, careful supervision and measures such as reducing the dose are recommended.

5. Use during Pregnancy, Delivery or Lactation

The safety of TJ-79 in pregnant women has not been established. Therefore, TJ-79 should be used in pregnant women, women who may possibly be pregnant only if the expected therapeutic benefits outweigh the possible risks associated with treatment.

6. Pediatric Use

The safety of TJ-79 in children has not been established. [Insufficient clinical data]

PACKAGING

Bottles of 500 g
2.5 g × 42 packets
2.5 g × 189 packets

REQUEST FOR LITERATURE SHOULD BE MADE TO:

Consumer Information Services Center
Tsumura & Co.
2-17-11 Akasaka, Minato-ku, Tokyo 107-8521, Japan

Manufactured and Distributed by:

Tsumura & Co.
2-17-11 Akasaka, Minato-ku, Tokyo 107-8521, Japan

Heiisan

TJ-79 平胃散 3D HPLC Pattern

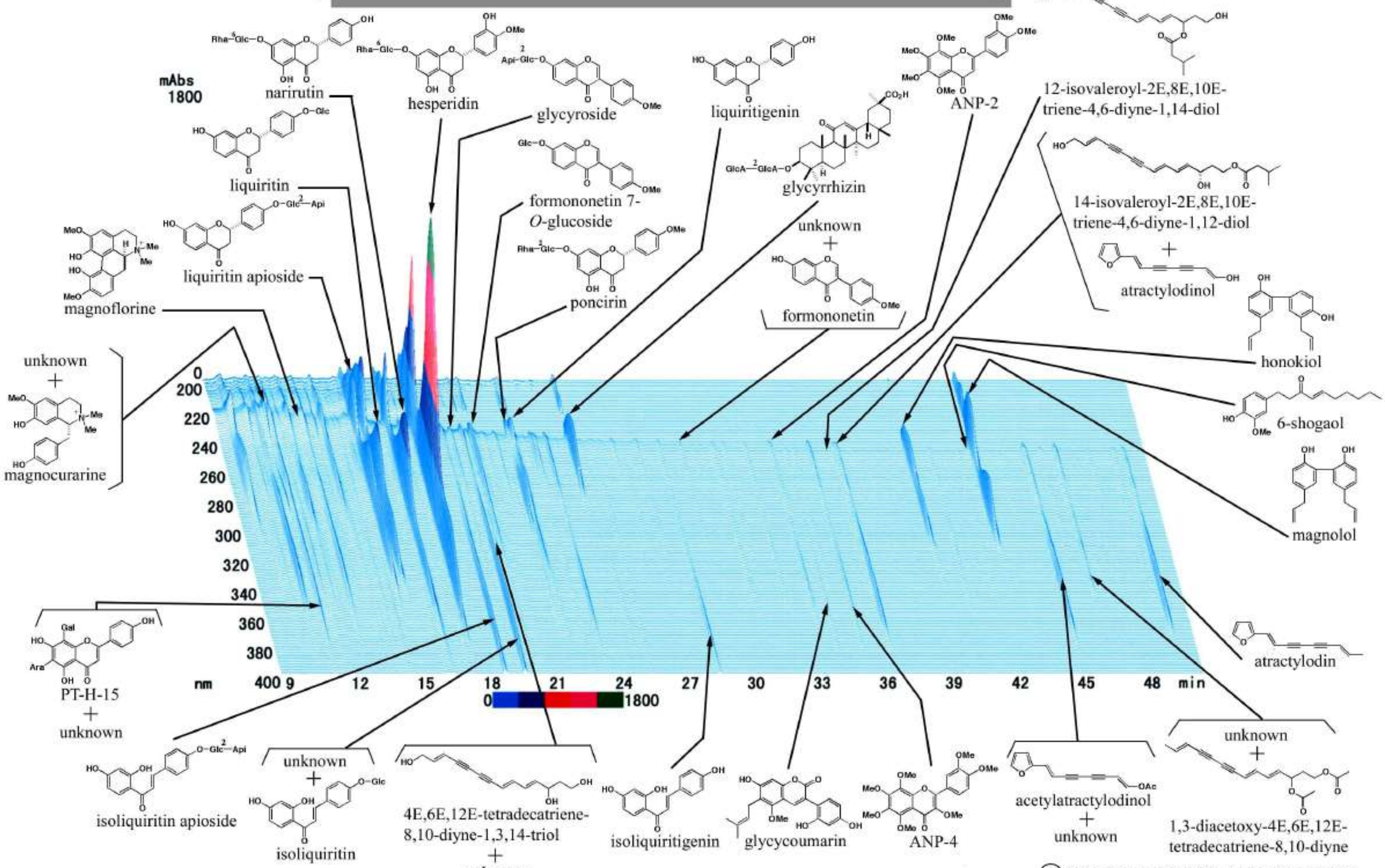

© 2001 TSUMURA & CO. All Rights Reserved.

Revised: March 2013 (5th version)

Standard Commodity Classification No. of Japan
875200

- Kampo-preparation-

TSUMURA Nichinto Extract Granules for Ethical Use

<nichinto>

Storage
Store in light-resistant, air-tight containers.

Expiration date
Use before the expiration date indicated on the container and the outer package.

Approval No.	(61AM)3291
Date of listing in the NHI reimbursement price	October 1986
Date of initial marketing in Japan	October 1986

DESCRIPTION

Composition	7.5 g of TSUMURA Nichinto extract granules contains 3.0 g of a dried extract of the following mixed crude drugs.<br>JP Pinellia Tuber .... 5.0 g<br>JP Poria Sclerotium .... 5.0 g<br>JP Citrus Unshiu Peel .... 4.0 g<br>JP Glycyrrhiza ….................................. 1.0 g<br>JP Ginger .... 1.0 g<br>(JP: The Japanese Pharmacopoeia)	
	Inactive ingredients	JP Magnesium Stearate<br>JP Lactose Hydrate<br>Sucrose Esters of Fatty Acids
Description	Dosage form	Granules
	Color	Light grayish-brown
	Smell	Characteristic smell
	Taste	Slightly sweet
	ID code	TSUMURA/81

INDICATIONS

TSUMURA Nichinto Extract Granules (hereafter TJ-81) is indicated for the relief of the following symptoms:
Nausea and vomiting

DOSAGE AND ADMINISTRATION

The usual adult dose is 7.5 g/day orally in 2 or 3 divided doses before or between meals. The dosage may be adjusted according to the patient's age and body weight, and symptoms.

PRECAUTIONS

1. Important Precautions

(1) When TJ-81 is used, the patient's "SHO" (constitution/symptoms) should be taken into account. The patient's progress should be carefully monitored, and if no improvement in symptoms/findings is observed, continuous treatment should be avoided.

(2) Since TJ-81 contains Glycyrrhiza, careful attention should be paid to the serum potassium level, blood pressure, etc., and if any abnormality is observed, administration should be discontinued.

(3) When TJ-81 is coadministered with other Kampo-preparations (Japanese traditional herbal medicines), etc., attention should be paid to the duplication of the contained crude drugs.

SHO: The term "SHO" refers to a particular pathological status of a patient evaluated by the Kampo diagnosis, and is patterned according to the patient's constitution, symptoms, etc. Kampo-preparations (Japanese traditional herbal medicines) should be used after confirmation that it is suitable for the identified "SHO" of the patient.

2. Drug Interactions

Precautions for coadministration (TJ-81 should be administered with care when coadministered with the following drugs.)

Drugs	Signs, Symptoms, and Treatment	Mechanism and Risk Factors
(1) Preparations containing Glycyrrhiza<br>(2) Preparations containing glycyrrhizinic acid or glycyrrhizinates	Pseudoaldosteronism is likely to occur. Besides, myopathy is likely to occur as a result of hypokalemia.<br>(Refer to the section "Clinically significant adverse reactions".)	Since glycyrrhizinic acid has an accelerating action on the potassium excretion at the renal tubules, an acceleration of decrease in the serum potassium level has been suggested.

3. Adverse Reactions

TJ-81 has not been investigated (drug use investigations, etc.) to determine the incidence of adverse reactions. Therefore, the incidence of adverse reactions is not known.

(1) Clinically significant adverse reactions

1) **Pseudoaldosteronism**: Pseudoaldosteronism such as hypokalemia, increased blood pressure, retention of sodium/body fluid, edema, increased body weight, etc. may occur. The patient should be carefully monitored (measurement of serum potassium level, etc.), and if any abnormality is observed, administration should be discontinued and appropriate measures such as administration of potassium preparations should be taken.

2) **Myopathy**: Myopathy may occur as a result of hypokalemia. The patient should be carefully monitored, and if any abnormality such as weakness, convulsion/paralysis of limbs, etc. are observed, administration should be discontinued and appropriate measures such as administration of potassium preparations should be taken.

4. Use in the Elderly

Because elderly patients often have reduced physiological function, careful supervision and measures such as reducing the dose are recommended.

5. Use during Pregnancy, Delivery or Lactation

The safety of TJ-81 in pregnant women has not been established. Therefore, TJ-81 should be used in pregnant women, women who may possibly be pregnant only if the expected therapeutic benefits outweigh the possible risks associated with treatment.

6. Pediatric Use

The safety of TJ-81 in children has not been established. [Insufficient clinical data]

PACKAGING

Bottles of 500 g
2.5 g × 42 packets
2.5 g × 189 packets

REQUEST FOR LITERATURE SHOULD BE MADE TO:

Consumer Information Services Center
Tsumura & Co.
2-17-11 Akasaka, Minato-ku, Tokyo 107-8521, Japan

Manufactured and Distributed by:

Tsumura & Co.
2-17-11 Akasaka, Minato-ku, Tokyo 107-8521, Japan

Nichinto

TJ-81 二陳湯 3D HPLC Pattern

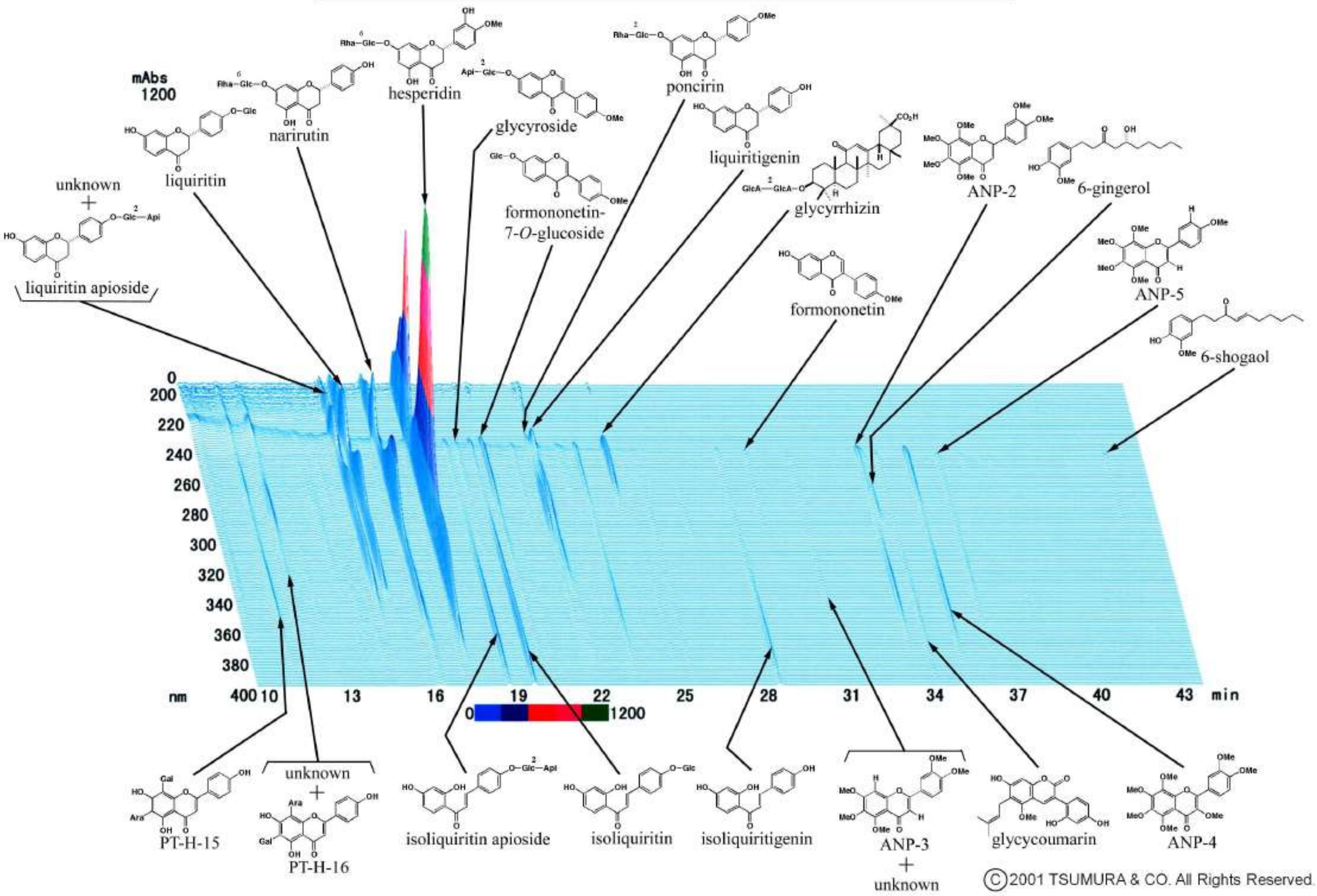

©2001 TSUMURA & CO. All Rights Reserved.
