# Supplementary material for: Exploratory assessment of the potential protective effects of Kampo treatment against low dose medical radiation: a single arm prospective observational study
Source: Front Pharmacol. 2026 Aug 7;17:1888108. doi: 10.3389/fphar.2026.1888108 (PMC13493526; doi:10.3389/fphar.2026.1888108)
Supplement: Supplementary file 2 [file Supplementaryfile3.pdf]

Supplementary Method

Human Primary Lymphocyte Culture

Blood samples were obtained prior to and following the commencement of treatment. Peripheral blood lymphocytes (PBLs) were isolated from the blood using Lymphoprepe™ (Axis-Shield PoC AS, Oslo, Norway) and cultured at 37°C in RPMI-1640 media supplemented with 20% fetal calf serum, 2% phytohemagglutinin (Remel Europe Ltd., Dartford, UK), and 0.05 μg/mL Colcemide™ (Gibcot®/Life Technologies, Grand Island, NY) in a 5% CO_2 atmosphere within a humidified incubator. After 48 hours of incubation, hypotonic treatment and fixation of lymphocytes were conducted according to established protocols (*38*). Slides with metaphase chromosomes were prepared using a HANABI Metaphase Spreader (ADSTEC Corp., Funabashi, Japan).

Telomere-Centromere Metaphase FISH

Metaphase FISH analysis was conducted following established protocols (*38*). Cy3-labeled centromere PNA probe (Cent Cy3, 5'-AAACTAGACAGAAGCATT-3') and FAM-labeled telomere PNA probe (TelC-FAM, 5'-CCCTAACCCTAACCCTAA-3'), both sourced from Panagene (Daejeon, South Korea), were utilized at a final concentration of 25 nmol/L. Following hybridization, the slides were mounted with Vectashield containing DAPI (Vectort Laboratories, Burlingame, CA).

Image Analyses

The slides were automatically imaged using a CoolCube 1 camera, equipped with MetaSystems (Altlussheim, Germany). Subsequently, chromosomal analysis was conducted using the Isis

imaging software (Meta-Systems), examining more than 2,000 and 1, 000 metaphase lymphocytes per sample in the control/CT scan groups and in the X-ray group, respectively. Metaphase lymphocytes exhibiting 46 centromere signals were recorded, while abnormal chromosomes displaying 'n' centromeres were categorized as 'n-1' dicentric chromosomes (*38*). All assessments were carried out in a blinded manner.

Immunofluorescent analyses of γ-H2AX foci

Cells were fixed in 4% paraformaldehyde and permeabilization with 0.5%Triton X-100, the cells were incubated with anti-γ-H2AX antibodies (1:1000; JBW301, Millipore, Billerica, MA, USA) at 37°C for 30 minutes. This was followed by another 30-minute incubation at 37°C with Cy3-conjugated secondary antibodies (1:200), cells were mounted using Vectashield containing DAPI (Vector Laboratories) and the images were acquired automatically with a fully motorized Axio Imager Z2 microscope (Carl Zeiss) equipped with the MetaSystems software (Altlussheim, Germany). The number of γ-H2AX foci in a mean of 3809 (range: 661–4168) cells in each specimen was automatically assessed. The number of γ-H2AX foci was normalized to the total cell count and expressed as average number of γ-H2AX foci per cell.
