# Supplementary material for: Exploratory assessment of the potential protective effects of Kampo treatment against low dose medical radiation: a single arm prospective observational study
Source: Front Pharmacol. 2026 Aug 7;17:1888108. doi: 10.3389/fphar.2026.1888108 (PMC13493526; doi:10.3389/fphar.2026.1888108)
Supplement: Supplementary file 3 [file Table1.docx]

**Supplementary Figure S1.** **Activation of human recombinant ALDH3A1 isozyme by D-limonene-containing Kampo formulas.** Bukuryoin, rikkunshito, heiisan and nichinto showed activation of ALDH3A1 enzyme activity of 217%, 200%, 244% and 183% over DMSO control (100%). Percent of enzyme activity is based on comparison with assays using DMSO as the control. Data are presented as means ± SD, n=3, * P<0.05, ** P<0.01.

**Supplementary Figure S2.** **Subgroup analysis of CAs induced by CT scans before and after Kampo treatment.**

(A) Changes in CAs by CT exposure before (left) and after (right) bukuryoin (befoe; p = 0.040, after; p = 0.735), (C) rikkunshito (before; p = 0.313, after; p = 0.625), (E) heiisan (before; p = 0.102, after; p = 0.898).(B) Comparison of CT-induced increase in CAs (exposed – unexposed) before and after bukuryoin (p = 0.244), (D) rikkunshito (p = 0.438), (F) heiisan (p = 0.413). Wilcoxon matched-pairs signed-rank test was used to compare each group. *p < 0.05; ns, not significant.

**Supplementary Figure S3.** **Subgroup analysis of 𝛾-H2AX foci induced by CT scans before and after Kampo treatment.**

(A) Changes in CAs by CT exposure before (left) and after (right) bukuryoin (before; p = 1.70×10^-2^, after; p = 4.46×10^-2^), (C) rikkunshito (before; p = 0.313, after; p = 0.844), (E) heiisan (before; p = 6.84×10^-3^, after; p = 6.74×10^-2^).(B) Comparison of CT-induced increase in CAs (exposed – unexposed) before and after bukuryoin (p = 0.847), (D) rikkunshito (p = 0.528), (F) heiisan (p = 5.37×10^-2^). Wilcoxon matched-pairs signed-rank test was used to compare each group. *p < 0.05; †p < 0.1; ns, not significant.
