# Supplementary material for: Exploratory assessment of the potential protective effects of Kampo treatment against low dose medical radiation: a single arm prospective observational study
Source: Front Pharmacol. 2026 Aug 7;17:1888108. doi: 10.3389/fphar.2026.1888108 (PMC13493526; doi:10.3389/fphar.2026.1888108)
Supplement: Supplementary file 4 [file Table2.docx]

**Supplementary Table S1. Baseline characteristics of participants according to Kampo formula subgroup.**

| **Variable** | **Bukuryoin** | **Rikkunshito** | **Heiisan** | **p-value** |
| --- | --- | --- | --- | --- |
|  |  |  |  |  |
| Age | 66(42.50-73.50) | 65.5(57.25-70.75) | 42(35.00-65.5) | 0.282 |
| Male/Female | 7/8 | 2/4 | 5/6 | 0.909 |
| Heigh (cm) | 160(154.80-167.5) | 159.8(151.90-171.75) | 163(155.35-164.90) | 0.974 |
| Weight (kg) | 56.2(50.75-64.40) | 46.5(40.93-55.00) | 58(53.10-67.50) | 0.227 |
| Baseline 𝛾-H2AX foci | 0.56(0.15-0.61) | 0.54(0.27-0.92) | 0.73(0.47-0.85) | 0.190 |

**Supplementary Table S2. CT exposure-related changes in CAs before Kampo treatment.**

| **Group** | **n** | **Unexposed** | **Before** | **Difference** | **p-value** |
| --- | --- | --- | --- | --- | --- |
|  |  |  | **Exposed** |  |  |
| All patients | 30 | 5.24 (3.65, 8.29) | 5.12 (3.53, 11.25) | 0.77 (−0.26, 3.47) | 0.023 |
| Kampo formula subgroup |  |  |  |  |  |
| Bukuryoin | 13 | 6.32 (3.80, 10.52) | 7.67 (4.96, 12.90) | 1.35 (0.39, 3.50) | 0.040 |
| Rikkunshito | 5 | 5.12 (4.08, 8.50) | 5.09 (1.93, 8.39) | –0.11 (−2.15, –0.03) | 0.313 |
| Heiisan | 11 | 4.64 (3.55, 6.81) | 4.89 (3.53, 11.25) | 2.92 (−0.52, 3.94) | 0.102 |
| Component subgroup |  |  |  |  |  |
| Citri Reticulatae Pericarpium |  |  |  |  |  |
| ≧3g | 25 | 5.37 (3.61, 7.67) | 5.15 (3.66 11.77) | 1.35 (−0.04, 3.90) | 0.007 |
| < 3g | 5 | 5.12 (4.08, 8.50) | 5.09 (1.93, 8.39) | –0.11 (−2.15, –0.03) | 0.313 |

Diff = median of exposed group − median of unexposed group.

**Supplementary Table S3. Median (IQR) of CT exposure-related changes in CAs before Kampo and after Kampo treatment.**

| **Group** | **n** | **Unexposed** | **After** | **Difference** | **p-value** |
| --- | --- | --- | --- | --- | --- |
|  |  |  | **Exposed** |  |  |
| All patients | 30 | 4.32 (2.61, 9.87) | 5.76 (3.24, 10.79) | 0.66 (–1.50, 1.52) | 0.428 |
| Kampo formula subgroup |  |  |  |  |  |
| Bukuryoin | 13 | 7.27 (2.61, 12.09) | 10.57 (4.64, 12.25) | 0.47 (–1.57, 1.56) | 0.735 |
| Rikkunshito | 5 | 2.99 (2.46, 6.58) | 5.96 (3.18, 8.30) | 0.72 (–1.05, 4.84) | 0.625 |
| Heiisan | 11 | 4.45 (3.14, 5.49) | 4.96 (3.26, 6.44) | 0.64 (–1.44, 1.35) | 0.898 |
| Component subgroup |  |  |  |  |  |
| Citri Reticulatae Pericarpium |  |  |  |  |  |
| ≧3g | 25 | 4.45 (2.61, 7.26) | 5.55 (3.43, 7.62) | 0.64 (–1.57, 1.41) | 0.615 |
| < 3g | 5 | 2.99 (2.46, 6.58) | 5.96 (3.18, 8.30) | 0.72 (–1.05, 4.84) | 0.625 |

Data are presented as median (IQR).

**Supplementary Table S4. Median (IQR) of CT exposure-related changes in number of 𝛾-H2AX foci before and after Kampo treatment.**

| **Group** | **n** | **Unexposed** | **Before** | **Difference** | **p-value** |
| --- | --- | --- | --- | --- | --- |
|  |  |  | **Exposed** |  |  |
| All patients | 33 | 0.58 (0.22, 0.81) | 0.68 (0.41, 1.09) | 0.18 (0.06, 0.29) | 2.97×10^-4^ |
| Kampo formula subgroup |  |  |  |  |  |
| Bukuryoin | 15 | 0.56 (0.15, 0.61) | 0.60 (0.31, 0.62) | 0.07 (0.04, 0.26) | 1.70×10^-2^ |
| Rikkunshito | 6 | 0.54 (0.27, 0.92) | 0.90 (0.52, 1.23) | 0.08 (0.21, 0.26) | 0.313 |
| Heiisan | 11 | 0.73 (0.47, 0.85) | 0.98 (0.61, 1.14) | 0.23 (0.12, 0.30) | 6.84×10^-3^ |
| Component subgroup |  |  |  |  |  |
| Citri Reticulatae Pericarpium |  |  |  |  |  |
| ≧3g | 27 | 0.58 (0.25, 0.77) | 0.68 (0.39, 1.04) | 0.14 (0.06, 0.29) | 5.02×10^-4^ |
| < 3g | 6 | 0.54 (0.27, 0.92) | 0.90 (0.52, 1.23) | 0.08 (0.21, 0.26) | 0.313 |

Data are presented as median (IQR).

**Supplementary Table S5. Median (IQR) of CT exposure-related changes in number of 𝛾-H2AX foci b after Kampo treatment.**

| **Group** | **n** | **unexposed** | **After** | **Difference** | **p-value** |
| --- | --- | --- | --- | --- | --- |
|  |  |  | **exposed** |  |  |
| All patients | 33 | 0.60 (0.34, 0.89) | 0.63 (0.38, 0.99) | 0.09 (–0.07, 0.08) | 1.89×10^-2^ |
| Kampo formula subgroup |  |  |  |  |  |
| Bukuryoin | 15 | 0.51 (0.25, 0.84) | 0.63 (0.39, 0.67) | 0.12 (–0.04, 0.26) | 4.46×10^-2^ |
| Rikkunshito | 6 | 0.73 (0.52, 0.88) | 0.63 (0.41, 0.92) | 0.02 (–0.13, 0.34) | 0.844 |
| Heiisan | 11 | 0.68 (0.42, 0.93) | 0.72 (0.47, 1.23) | 0.09 (0.02, 0.20) | 6.74×10^-2^ |
| Component subgroup |  |  |  |  |  |
| Citri Reticulatae Pericarpium |  |  |  |  |  |
| ≧3g | 27 | 0.51 (0.34, 0.90) | 0.63 (0.39, 1.01) | 0.09 (0.01, 0.26) | 7.66×10^-3^ |
| < 3g | 6 | 0.73 (0.52, 0.88) | 0.63 (0.41, 0.92) | 0.02 (–0.13, 0.34) | 0.844 |

Data are presented as median (IQR).
